# A histone-mimicking interdomain linker in a multidomain protein modulates multivalent histone binding

**DOI:** 10.1074/jbc.M117.801464

**Published:** 2017-09-01

**Authors:** Sebastian Kostrhon, Georg Kontaxis, Tanja Kaufmann, Erika Schirghuber, Stefan Kubicek, Robert Konrat, Dea Slade

**Affiliations:** From the ‡ Department of Biochemistry, Max F. Perutz Laboratories, University of Vienna, Campus Vienna Biocenter, Dr. Bohr-Gasse 9, 1030 Vienna, Austria,; the § Department of Structural and Computational Biology, Max F. Perutz Laboratories, University of Vienna, Campus Vienna Biocenter 5, 1030 Vienna, Austria,; the ¶ CeMM Research Center for Molecular Medicine of the Austrian Academy of Sciences, 1030 Vienna, Austria, and; the ‖ Christian Doppler Laboratory for Chemical Epigenetics and Antiinfectives, CeMM Research Center for Molecular Medicine of the Austrian Academy of Sciences, 1030 Vienna, Austria

**Keywords:** histone, intrinsically disordered protein, isothermal titration calorimetry (ITC), NMR, PHD finger, posttranslational modification (PTM), BAZ2B, bromodomain, multivalent binding

## Abstract

N-terminal histone tails are subject to many posttranslational modifications that are recognized by and interact with designated reader domains in histone-binding proteins. BROMO domain adjacent to zinc finger 2B (BAZ2B) is a multidomain histone-binding protein that contains two histone reader modules, a plant homeodomain (PHD) and a bromodomain (BRD), linked by a largely disordered linker. Although previous studies have reported specificity of the PHD domain for the unmodified N terminus of histone H3 and of the BRD domain for H3 acetylated at Lys^14^ (H3K14ac), the exact mode of H3 binding by BAZ2B and its regulation are underexplored. Here, using isothermal titration calorimetry and NMR spectroscopy, we report that acidic residues in the BAZ2B PHD domain are essential for H3 binding and that BAZ2B PHD–BRD establishes a polyvalent interaction with H3K14ac. Furthermore, we provide evidence that the disordered interdomain linker modulates the histone-binding affinity by interacting with the PHD domain. In particular, lysine-rich stretches in the linker, which resemble the positively charged N terminus of histone H3, reduce the binding affinity of the PHD finger toward the histone substrate. Phosphorylation, acetylation, or poly(ADP-ribosyl)ation of the linker residues may therefore act as a cellular mechanism to transiently tune BAZ2B histone-binding affinity. Our findings further support the concept of interdomain linkers serving a dual role in substrate binding by appropriately positioning the adjacent domains and by electrostatically modulating substrate binding. Moreover, inhibition of histone binding by a histone-mimicking interdomain linker represents another example of regulation of protein–protein interactions by intramolecular mimicry.

## Introduction

Posttranslational modifications (PTMs)^3^ of histones modulate chromatin-templated biological processes such as transcription and DNA replication and repair by modifying local chromatin structure and composition (1–5). Histone PTMs can directly alter the physical properties of individual nucleosomes by modulating electrostatic histone–DNA and histone–histone interactions or indirectly by recruiting effector proteins, which contain specific PTM recognition domains (1, 6, 7). By binding single or multiple histone marks, effector proteins can cross-link two or more nucleosomes (8, 9), increase the presence of the RNA polymerase complex and related factors (10), or recruit ATP-dependent remodeling complexes (11, 12).

Histone PTMs can be bound by a large number of specialized recognition domains in effector proteins (6, 7). Acetylation marks are read by bromodomain (BRD) and plant homeodomain (PHD) fingers, whereas methylation is recognized by the chromodomain, the Tudor domain, BAH (Bromo-adjacent homology) domains, MBT (malignant brain tumor) repeats, ADD (ATRX-DNMT3-DNMT3L) domains, WD40 repeats, PWWP, and PHD fingers (6, 7). Although many chromatin-associated proteins encode only a single reader module, a significant number of effector proteins contain multiple chromatin recognition domains (13, 14). Compared with monovalent interactions, multivalent binding of multiple histone PTMs dramatically enhances specificity and affinity through additive enthalpies of each binding event and reduced entropy loss (13). In *cis* and in *trans* binding occur when one multivalent reader protein (or an effector complex) binds histone modifications on the same or on different histones, respectively (15, 16).

In multidomain proteins, modular domains are often connected by flexible linkers that are composed of intrinsically disordered regions (IDRs) (17). IDRs are characterized by low sequence complexity and a high proportion of charged and hydrophilic amino acids (17). The physical and chemical properties of intrinsically disordered linkers modulate the interdomain distance and the relative multidomain orientation as well as substrate-binding affinity, protein activity, and protein–protein interactions (18, 19). For example, a polybasic linker at the C terminus of the ubiquitin ligase UHRF1 allosterically abrogates binding of H3K9me3 to the tandem Tudor domain (20). As a possible regulatory mechanism, binding of the negatively charged phosphatidylinositol 5-phosphate to the polybasic linker alleviates the inhibition by inducing a conformational change (20).

The human BROMO domain adjacent to zinc finger 2B (BAZ2B) protein is a multidomain histone-binding protein that contains a PHD zinc finger and a BRD domain connected via a 72-amino acid linker. In addition, BAZ2B contains several DNA-binding domains, such as an MBD (methyl-CpG–binding domain), AT hook, and DDT (DNA binding homeobox and different transcription factors). It shares 29% sequence identity with BAZ2A, also called TIP5 (transcription termination factor I-interacting protein 5), which is part of the nucleolar remodeling complex (21). BAZ2A binds to silent rRNA genes after passage of the replication fork and recruits chromatin modifiers to re-establish silent chromatin (21, 22). However, other than the recent structural analysis of BRD–H3K14ac binding (23) and PHD–H3 binding (24), very little is known about BAZ2B. The BAZ2B PHD finger assumes a canonical “cross-braced topology,” where two Zn^2+^ ions are coordinated by Cys^4^-His-Cys^3^ (23). Residues 1943–1945 form a right-handed 3_10_ helix followed by two antiparallel β-strands. The N-terminal residues of unmodified H3 peptide (H3(1–4)) form the third antiparallel β-strand when bound to the PHD domain, whereas subsequent H3 residues are folded to an α-helix (23, 24). In contrast to the PHD domains of BPTF (bromodomain PHD finger transcription factor) (25) and PHF2 (PHD finger protein 2) (26), the BAZ2B PHD domain lacks an aromatic cage that would allow hydrophobic interactions with the methyl group of Lys^4^. However, the BAZ2B PHD can still bind H3K4(me1–3), albeit with reduced affinity (23). The BAZ2B BRD forms a typical bromodomain fold with four helices (αZ, αA, αB, and αC) connected by three loops (AB, ZA, and BC), which form a binding groove for the K14ac side chain (23). The interaction between the conserved Asn^2140^ in the BC loop and K14ac is additionally stabilized through hydrogen bonds with the H3 backbone.

The charged linker between the PHD and BRD of BAZ2B is difficult to map structurally using X-ray crystallography (23), which indicates that the linker region is disordered and flexible. Furthermore, close inspection of the positively charged lysine-arginine–rich linker revealed some resemblance to the histone substrate (Fig. 4*B*), which raises the possibility of competitive interaction of the linker with the PHD/BRD. Hence, the main aim of this work was to investigate the importance of the size and composition of the BAZ2B linker for binding of the N-terminal histone H3 tail to BAZ2B. Using isothermal titration calorimetry (ITC) and NMR spectroscopy, we show that mutation or deletion of the conserved parts of the BAZ2B linker alters H3-binding affinities due to of changes in its charge. Surprisingly, removal of the positively charged residues or introduction of the negative charge via phosphomimicking mutations increases BAZ2B-binding affinity toward the H3 substrate. This points to an active role of the linker in regulating substrate binding to the active site. Phosphorylation, acetylation, or poly(ADP-ribosyl)ation of the BAZ2B linker may adjust the linker net charge *in vivo* and thereby regulate the multivalent binding of the BAZ2B PHD and BRD to histone substrates.

## Results

### Acidic residues in the PHD domain regulate H3 binding

**Figure 1.**
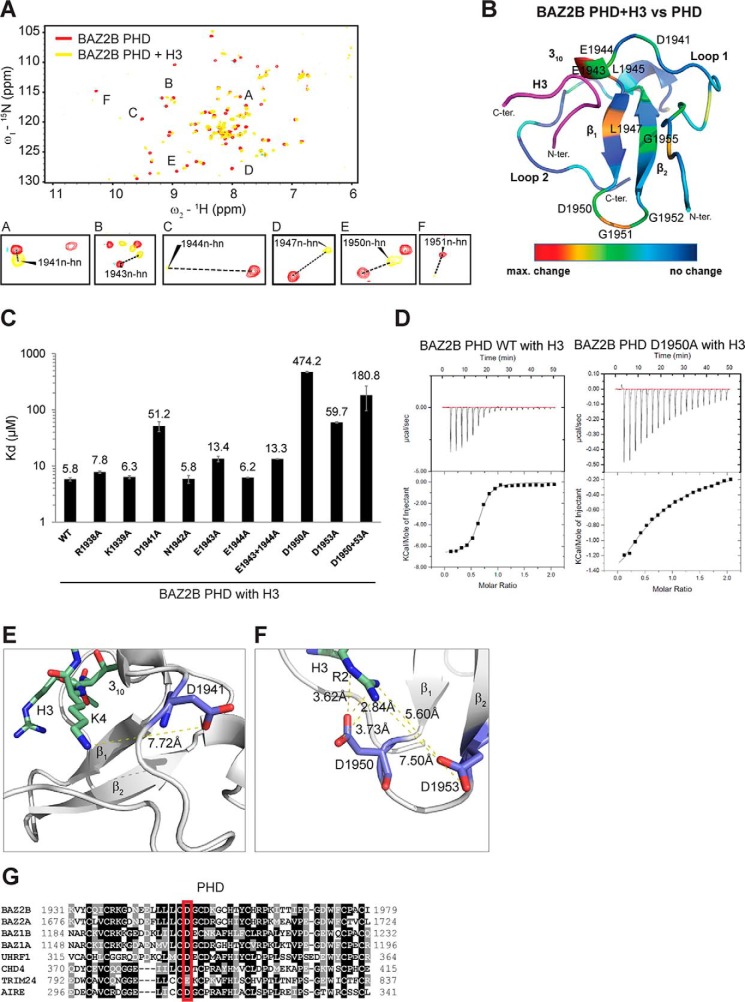
**Negatively charged residues in the BAZ2B PHD bind histone H3.** *A*, a shift difference map deduced from ^15^N HSQC spectra of the ^15^N-labeled BAZ2B PHD zinc finger in the absence and presence of H3(1–21aa)K4. *B*, differences in shifts between the native BAZ2B PHD and upon binding the unmodified H3(1–21aa) peptide from *A* are mapped onto the BAZ2B PHD zinc finger (PDB code 4QF3) and visualized as color coding using PyMOL (*red*, maximum (*max*) changes; *blue*, no shifts). *N-ter*, N terminus; *C-ter*, C terminus. *C*, comparison of binding affinities of BAZ2B PHD WT and various mutants. *Error bars* represent mean ± S.D. (*n* = 4). *D*, ITC binding curves of PHD WT and PHD D1950A binding to H3(1–21aa). *E*, Asp^1941^ forms electrostatic interactions with H3K4 (PDB code 4Q6F). *F*, Asp^1950^ and Asp^1953^ form an acidic cavity for H3R2 binding (PDB code 4Q6F). *G*, conserved residues across different PHD fingers. The sequences correspond to AIRE_1 (O43918 AIRE_HUMAN), BAZ1A (Q9NRL2 BAZ1A_HUMAN), BAZ1B (Q9UIG0 BAZ1B_HUMAN), BAZ2A (Q9UIF9 BAZ2A_HUMAN), BAZ2B (Q9UIF8 BAZ2B_HUMAN), CHD4 PHD1 (Q14839 CHD4_HUMAN), and TRIM24 (O15164 TIF1α_HUMAN), UHRF1 (Q96T88 UHRF1_HUMAN).

^15^N HSQC spectra of uniformly ^15^N-labeled BAZ2B PHD domain or PHD–BRD were recorded in the presence of unmodified H3(1–21) (Fig. 1, *A* and *B* and supplemental Fig. 1*A*). When comparing the chemical shifts of amino acids in the spectra of the native PHD (without the peptide) and PHD bound to H3, we confirmed involvement of the residues in the 3_10_ helix and along the β1-strand in the binding of H3 (Fig. 1*A*). The amplitude of changes of ^1^H and ^15^N chemical shifts were color-coded from *blue* (lowest) to *red* (highest) and mapped onto the BAZ2B PHD structure (for the BAZ2B PHD in Fig. 1*B* and the BAZ2B PHD–BRD in supplemental Fig. 1*A*). Residues that exhibited the strongest chemical shift changes upon H3 binding to the BAZ2B PHD were Glu^1943^, Glu^1944^, Leu^1945^, Leu^1947^, Asp^1950^, and Gly^1951^ (Fig. 1, *A* and *B*). They were already implicated in H3 binding based on the BAZ2A PHD-H3 structures and ^15^N HSQC spectra as well as BAZ2B PHD-H3(1–5aa) ^15^N HSQC spectra (23, 24). Glu^1944^, Leu^1945^, and Gly^1951^ reproducibly showed maximum shifts in the context of BAZ2B PHD–BRD binding to H3 (supplemental Fig. 1*A*).

To investigate the contribution of amino acids in the PHD that exhibited chemical shifts upon H3 binding, we introduced a series of point mutations in the PHD and PHD–BRD constructs and measured their effects on H3-binding affinity by ITC (Fig. 1, *C* and *D*, and supplemental Fig. 1*B*). The mutation sites were selected based on the proximity to H3 residues and their conservation in similar PHD fingers (Fig. 1*G*). The BAZ2B PHD bound H3(1–21aa) with *K_d_* = 5.78 μm (Fig. 1, *C* and *D*).

Notwithstanding the observed chemical shifts, the E1944A mutation had no effect on H3-binding affinity, whereas the E1943A mutation led to a 2-fold reduction (Fig. 1*C*). The D1941A mutation led to a 10-fold decrease in binding affinity, which may be due to the loss of electrostatic interactions with H3K4 and loss of H-bonding with the H3 backbone (Fig. 1*E*). Notably, residues Asp^1950^ and Asp^1953^ showed a 100- and 10-fold reduction in binding affinity, respectively (Fig. 1, *C* and *D*). Asp^1950^ and Asp^1953^ form an acidic cavity for hydrogen bonding with the guanidine nitrogen (Nη) atoms of Arg^2^ (Fig. 1*F*). Asp^1950^ is conserved in different PHD fingers, which points to its important role in histone H3 binding (Fig. 1*G*). The results of this extensive mutational analysis of BAZ2B PHD–H3 binding suggest that negatively charged residues such as Asp^1941^, Asp^1950^, and Asp^1953^ are important for binding the positively charged residues (Arg^2^ and Lys^4^) in the N-terminal part of the H3 peptide (Fig. 1*C* and supplemental Fig. 1*B*).

### Histone H3 makes extensive contacts with the PHD

**Figure 2.**
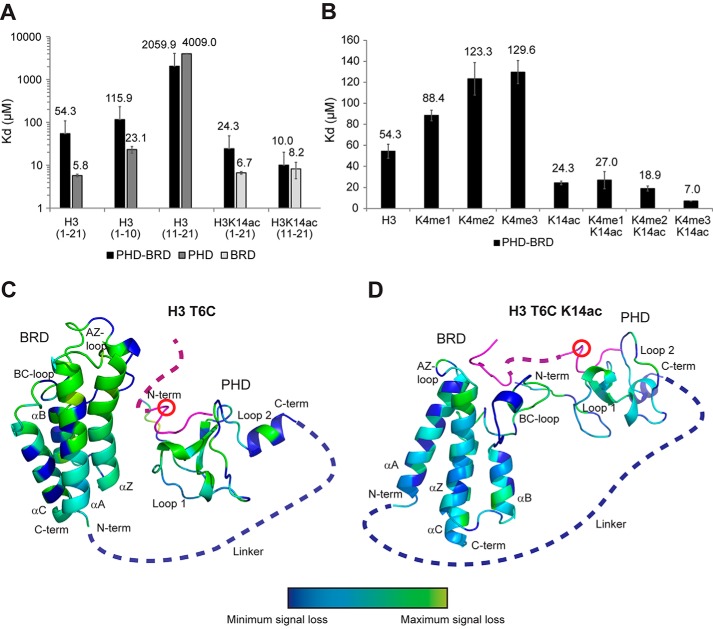
**Combined readout of H3K4K14ac by BAZ2B PHD–BRD.** *A*, the binding affinity of BAZ2B PHD, BRD, and PHD–BRD toward H3(1–10aa), H3(11–21aa), H3(1–21aa), H3(11–21aa)K14ac, and H3(1–21aa)K14ac, measured by ITC. *Error bars* represent mean ± S.D. (*n* = 4). *B*, the binding affinity of BAZ2B PHD–BRD toward H3K4(me0–3)K14(ac), measured by ITC. *Error bars* represent mean ± S.D. (*n* = 4). *C* and *D*, loss of peak intensities, measured by paramagnetic relaxation enhancement upon binding of MTSL-tagged H3T6C (*C*) and H3T6CK14ac (*D*) to BAZ2B PHD–BRD. The position of the MTSL label is marked with a *red circle*. Loss of peak intensities is displayed on the BAZ2B PHD–BRD model based on the color coding: *blue*, native; *cyan*, small change; *green*, intermediate change; *yellow*, maximum loss of peak intensity. The BAZ2B PHD–BRD model was created using the already published crystallography data from BAZ2B PHD native (PDB code 4QF3), BAZ2B BRD with H3K14ac (PDB code 4QC1), and BAZ2A (TIP5) with H3(1–10aa) (PDB code 5T8R) as a template. *N-ter*, N terminus; *C-ter*, C terminus.

Having found the most important residues of the PHD finger involved in H3 binding, we next sought to get more detailed insights into the residues of the H3 peptide that are bound by the PHD. We carried out ITC measurements of truncated unmodified or K14ac peptides (Fig. 2*A*). We observed specificity of the PHD finger toward H3(1–10aa) (*K_d_* = 23.13 μm) compared with *K_d_* > 4 mm for H3(11–21aa) (Fig. 2*A*). The same is true for the PHD–BRD construct (115 μm for H3(1–10aa) compared with >2 mm for H3(11–21aa)) (Fig. 2*A*). However, both PHD and PHD–BRD bound H3(1–21aa) with 2- to 4-fold higher affinity than H3(1–10aa), which indicates that H3 makes additional contacts with the PHD beyond the first 10 residues (Fig. 2*A*). We did not observe the same effect when comparing BRD binding to H3K14ac(1–21aa) *versus* H3K14ac(11–21aa) (Fig. 2*A*), as BRD binding relies on the K14ac-*X-X*-Arg^17^ motif (23), making it independent of residues 1–10 of H3. A 4-fold increased affinity of the PHD toward H3(1–10aa) compared with H3(1–5aa) was recently explained by the intramolecular stabilization of the H3 helical fold based on the co-structure of BAZ2A PHD-H3(1–10aa) (24).

### Combined readout of H3K14ac by BAZ2B PHD–BRD

We observed a significant difference in binding affinity when comparing affinity constants for BAZ2B BRD and PHD–BRD toward H3(1–21aa)K14ac or BAZ2B PHD–BRD toward H3(1–21aa)K14ac and H3(11–21aa)K14ac (Fig. 2*A*). The affinity of BAZ2B PHD-BRD toward H3(1–21aa)K14ac was 3.6-fold reduced compared with BAZ2B BRD and 2.4-fold reduced compared with H3(11–21aa)K14ac. These results indicate that the PHD linker domain negatively modulates the binding of the BRD to H3K14ac, suggesting that the two domains act in a combined manner to bind H3K14ac.

To investigate this further, we compared the binding affinity of BAZ2B PHD–BRD to H3K4(me0–3)K14(ac) peptides (Fig. 2*B*). Addition of methyl groups at Lys^4^ progressively reduced the binding affinity, in agreement with the published data using BAZ2B PHD (23). Interestingly, K4me2/3 in combination with K14ac increased the binding affinity of PHD–BRD to the level of the BRD alone (Fig. 2, *A* and *B*). Given that binding of H3K4 to the PHD reduces the overall binding affinity of PHD–BRD to H3K14ac (Fig. 2*A*), whereas lack of binding of H3K4me3 to the PHD increases it (Fig. 2*B*), we conclude that the PHD and BRD bind H3K4K14ac in a multivalent manner in *cis*.

We next probed the relative orientation of the two domains when binding unmodified H3 and H3K14ac peptide by paramagnetic relaxation enhancement measurements. To this end, we replaced Thr^6^ in the H3(1–21aa) and H3(1–21aa)K14ac peptides with a cysteine and chemically labeled it with the nitroxide spin label MTSL, which results in greatly enhanced NMR relaxation for nearby nuclei (supplemental Fig. 2). The intensity ratios between the oxidized (paramagnetic MTSL-tagged) and reduced (diamagnetic) BAZ2B-H3(1–21aa)K14(ac) complexes are displayed in Fig. 2, *C* and *D*, and supplemental Fig. 2. The measurements showed loss of peak intensities near the binding site in the PHD (as expected) as well as losses in the linker close to the BRD and within the BRD itself. The losses were more pronounced for H3, which is not bound by the BRD, suggesting that, in the absence of tandem binding, the BRD still forms transient encounters with the PHD (Fig. 2, *C* and *D*). Upon tandem binding of H3K4K14ac, the shortest distance of encounter is limited by the H3 stretch between H3T6 and H3K14 (about 35 Å), and the PHD–BRD complex assumes a more extended conformation (Fig. 2, *C* and *D*). This is in line with the previously published SAXS data comparing PHD–BRD and PHD–BRD in complex with H3K14ac, which showed an increase in radius of gyration and *D*_max_ (maximum diameter) indicating a more extended conformation of the histone peptide complex (23). Our data also indicate that histone binding leads to, on average, a more extended conformation of PHD–BRD due to an H3K14ac-induced reduction in conformational space.

### The linker region between the BAZ2B PHD and BRD modulates H3-binding affinity by interacting with the PHD

**Figure 3.**
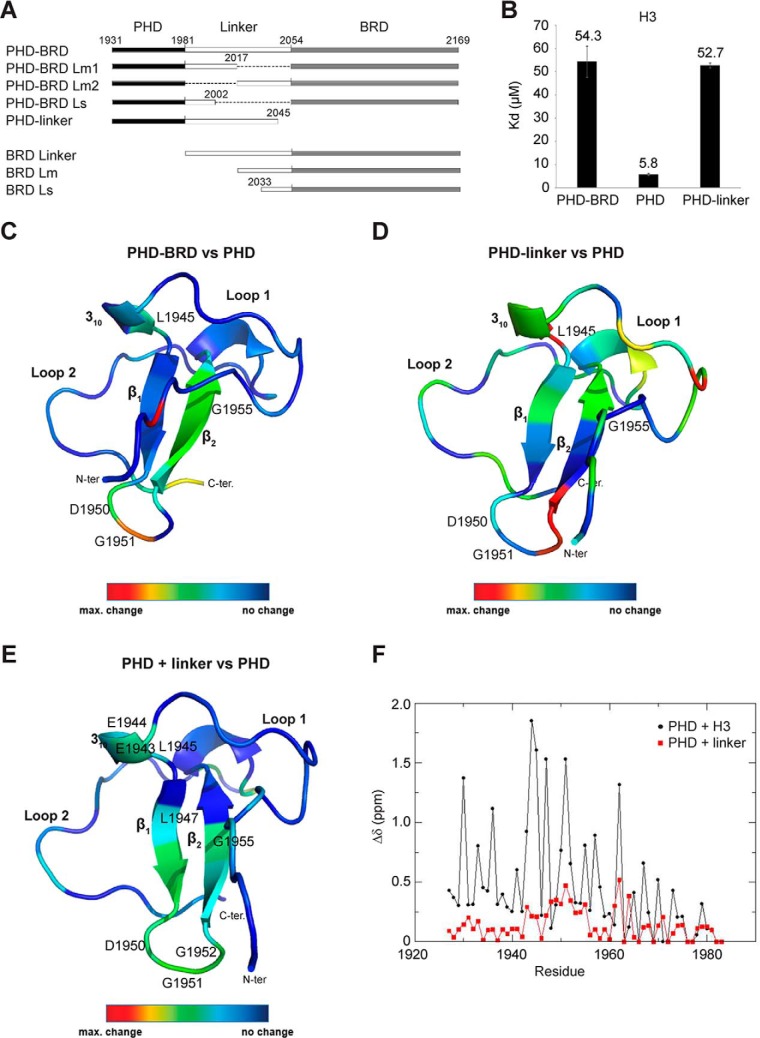
**The interdomain linker modulates the binding affinity of the BAZ2B PHD toward histone H3 by binding to the PHD.** *A*, schematic of all used BAZ2B constructs. *B*, binding affinities of BAZ2B PHD, the PHD linker, and PHD–BRD toward H3, measured by ITC. *Error bars* represent mean ± S.D. (*n* = 4). *C* and *D*, differences in chemical shifts between BAZ2B PHD–BRD and the PHD (*C*) and between the PHD linker and the PHD (*D*) visualized with a color code. *N-ter*, N terminus; *C-ter*, C terminus. *E*, differences in shifts between the native BAZ2B PHD domain and upon binding of the PHD-proximal linker peptide visualized with a color code. *F*, chemical shift perturbations (Δδ) for each residue of the ^15^N-labeled BAZ2B PHD between apo and bound spectra of PHD+H3(1–21aa) (*black*) and PHD+linker (^1982^ASGQTLKIKKLHVKGKKTNESKKGKK^2007^) (*red*).

The PHD domain showed a 9-fold higher affinity for H3(1–21aa) compared with the PHD–BRD construct (Fig. 2*A*), which suggests a modulatory effect of either the linker between the two domains or the BRD itself on PHD–H3 binding. To gain more insight into the underlying molecular mechanism, we compared the H3-binding affinity of BAZ2B PHD, PHD linker, and PHD–BRD (Fig. 3, *A* and *B*). ITC showed that the PHD linker construct has a very similar dissociation constant compared with the PHD–BRD construct (PHD–BRD = 54 μm; PHD linker = 52 μm), implicating the linker region itself as the main negative modulator of the binding affinity (Fig. 3*B*).

Analogously, NMR experiments provided further evidence that the linker induces changes in the spectra of the PHD (β_2_-strand) when comparing (^1^H and ^15^N) chemical shift differences between BAZ2B PHD and PHD–BRD as well as PHD and PHD linker spectra (Fig. 3, *C* and *D*, and supplemental Fig. 3, *A* and *B*). In particular, Asp^1950^, known to be crucial for H3 binding, showed pronounced signal shifts (Figs. 1*C* and 3, *C* and *D*). To test whether the linker can interact with the PHD in *trans*, we added a linker peptide (Ala^1982^-Lys^2007^aa) to the ^15^N-labeled PHD and observed chemical shift perturbations in the PHD, indicating that the linker can weakly but specifically bind the PHD domain (Fig. 3, *E* and *F*). Although the binding mode of the linker is not identical to H3, Asp^1950^ is strongly involved in both cases (Fig. 3*F*). Collectively, these data indicate transient binding of the linker to the PHD, giving rise to competition between the linker and the H3 peptide for binding to the PHD.

Considering that, in the case of multivalent interactions, ITC measurements provide only an average of binding events, we used NMR to examine the binding affinities of PHD and BRD residues for H3K14ac. We performed titration of H3K14ac into ^15^N-labeled PHD–BRD at the following peptide/protein ratios: 0.5, 1, 1.5, 2, and 4 (supplemental Fig. 4). Chemical shift perturbations in BRD residues followed a single-site binding model with saturation reached at 1:1 ratio and estimated *K_d_* > 8 μm (supplemental Fig. 4*B*). The *K_d_* values determined in this experiment agree reasonably well with the ITC measurement, given the limitations due to the concentration range required for NMR. Shifts in the PHD showed more complex binding and reached saturation at 1.5 ratio with an estimated *K_d_* > 190 μm (supplemental Fig. 4*C*). This confirms the autoinhibitory effect of the linker on H3 binding to the PHD.

Finally, we investigated whether the linker has a similar effect on the binding affinity of the BRD by generating constructs with different N-terminally truncated linkers (Fig. 3*A*). We did not observe an effect of the linker on BRD-binding affinity toward H3K14ac (supplemental Fig. 5), which confirms that the PHD linker domain, and not the linker *per se*, exerts negative cooperativity on BRD binding to H3K14ac.

### The linker length and amino acid composition influence H3 binding

**Figure 4.**
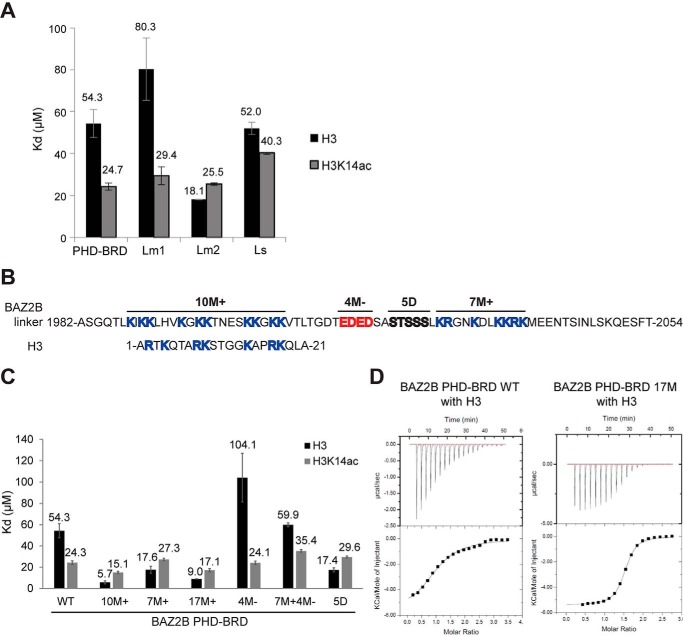
**Positively and negatively charged residues in the linker modulate the binding affinity of BAZ2B PHD toward H3.** *A*, binding affinities of BAZ2B PHD–BRD truncation constructs toward H3 and H3K14ac peptides, determined by ITC. *Error bars* represent mean ± S.D. (*n* = 4). *B*, the sequence of the BAZ2B linker resembles histone H3. Mutations of different regions in the BAZ2B linker are indicated at the *top. C*, binding affinities of the PHD–BRD bearing different linker mutations toward H3 and H3K14ac peptides, as determined by ITC. *Error bars* represent mean ± S.D. (*n* = 4). *D*, ITC binding curves for complex formation between BAZ2B PHD–BRD WT or PHD–BRD 17M and H3(1–21aa).

The 72 aa–long linker comprises two prominent stretches enriched in lysine and arginine residues, which are located in the PHD- and BRD-proximal part of the linker. We deleted the BRD-proximal part (BAZ2B PHD–BRD Lm1; linker length, 36 aa) as well as the PHD-proximal part of the linker (BAZ2B PHD–BRD Lm2; linker length, 36 aa) (Fig. 3*A*). ITC showed a lower H3-binding affinity of Lm1 (80 μm) and a higher affinity of Lm2 (18 μm) (Fig. 4*A*). This indicates that the PHD-proximal part of the linker competes with the PHD for H3 binding and that the BRD-proximal part of the linker somewhat neutralizes the inhibitory effects of the PHD-proximal part. Conversely, Lm1 and Lm2 bind H3K14ac with the same affinity as the wild-type BAZ2B PHD–BRD (Fig. 4*A*), which confirms that the linker does not interfere with the binding of K14ac to the BRD. Reduction of the linker length to 21 aa (BAZ2B Ls) reduced binding affinity for H3K14ac (Fig. 4*A*), which indicates the requirement of a minimal length and flexibility of the linker to prevent a steric clash between the PHD and BRD and allow their optimal mutual positioning for histone binding.

The BAZ2B linker is rich in basic amino acids, especially lysines, which are conserved across species (supplemental Fig. 6*A*). Moreover, charged amino acid motifs between histone recognition domains were also identified in related proteins such as UHRF1, TRIM24 (tripartite motif 24), and TRIM33 (tripartite motif 33) (27). Strikingly, the charged amino acid motifs within the BAZ2B linker have a net charge comparable with the H3(1–21aa) peptide (Fig. 4*B*). Mutation of the charged residues in the linker region led to a drastic change in H3-binding affinity (Fig. 4*C*). Alanine mutation of the PHD-proximal positively charged patch (10M^+^) and the BRD-proximal positively charged patch (7M^+^) led to a 9-fold and 3-fold increase in binding affinity toward H3, respectively (Fig. 4*C*). Combined mutation of 10M^+^ and 7M^+^ (17M^+^) did not increase the H3-binding affinity beyond that of 10M+ (Fig. 4, *C* and *D*).

Although the mutation of positively charged residues (7M^+^ or 10M^+^) increased the binding affinity, mutation of negatively charged residues led to a 2-fold reduction in binding affinity (Fig. 4*C*). The combined mutation of 7M^+^ and negatively charged residues (7M^+^ 4M^−^) restored the wild-type binding affinity (Fig. 4*C*). The same changes in H3-binding affinity were observed when all of the different mutations were introduced in the PHD linker construct (supplemental Fig. 6*B*). Given that 17M^+^ has a similar binding affinity as 10M^+^ and that mutations of negatively charged residues neutralize the 7M^+^ effect on binding affinity, we conclude that the positively charged residues in the PHD-proximal part of the linker are the primary contribution to the inhibitory effect of the linker on H3-binding affinity.

We additionally mutated several phosphorylatable residues (residues 2021–2025; 5D) present in the BRD-proximal part of the linker into phosphomimicking Asp residues (Fig. 4, *B* and *C*). Additional negative charges led to an increase in affinity and completely neutralized the inhibitory effect of the positively charged residues in the BRD-proximal part of the linker (Fig. 4*C*).

Furthermore, we explored the effect of the PHD-proximal part of the linker on the BRD by comparing chemical shift differences between BAZ2B BRD, PHD–BRD, and PHD–BRD 10M^+^ (supplemental Fig. 7). The BRD spectra differed from PHD–BRD WT but aligned well with PHD–BRD 10M^+^, indicating a transient interaction of the PHD-proximal part of the linker with the BRD. This indicates the importance of the linker in modulating not only the binding affinity of the PHD but also the relative positioning of the two domains.

### BAZ2B PHD–BRD binds H3K14ac in vivo

**Figure 5.**
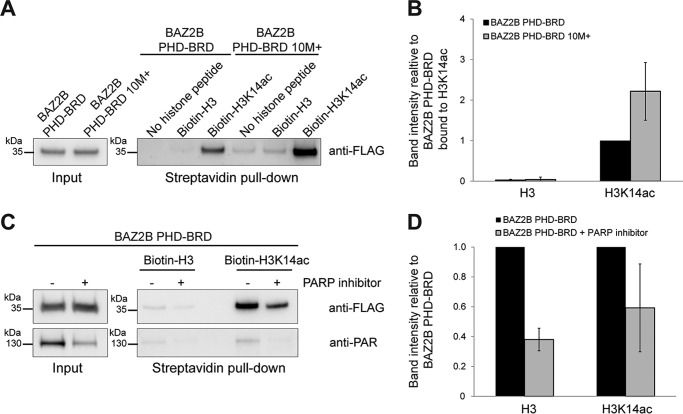
**BAZ2B PHD–BRD binds H3K14ac *in vivo*.** *A* and *C*, histone binding assay with biotinylated H3 and H3K14ac peptides, which were incubated with nuclear extracts from HEK293T cells transiently expressing Flag-BAZ2B PHD–BRD wild-type and the 10M^+^ mutant (*A*) or FLAG-BAZ2B PHD–BRD wild-type without or with treatment of cells with the PARP inhibitor olaparib at 1 μm during the 48 h of transfection (*C*). 0.5% of input and 30% of eluate were loaded on the gels. *B* and *D*, quantification of Western blots in *A* and *C. Error bars* represent mean ± S.D. (*n* = 3).

Changes in the linker net positive charge may occur *in vivo* by posttranslational modifications, whereby lysine acetylation or poly(ADP-ribosyl)ation reduce the positive charge and phosphorylation increases the negative charge. Given the effect of the electrostatic properties of the linker on histone binding, we tested whether BAZ2B PHD–BRD is also able to bind H3K14ac in the cellular environment. To this end, we performed a histone peptide binding assay using biotinylated peptides prebound to streptavidin beads and incubated with nuclear extracts from FLAG-BAZ2B PHD–BRD–transfected HEK293T cells (Fig. 5). Bound proteins were analyzed by Western blotting. We confirmed preferential binding of BAZ2B PHD–BRD to H3K14ac (Fig. 5, *A* and *B*). The 10M^+^ mutant exhibited 2- to 3-fold stronger binding to H3K14ac compared with the WT, corroborating *in vitro* ITC data (Figs. 5*B* and 4*C*). Reduction of cellular PAR levels with the PARP1/2 inhibitor olaparib reduced the binding of BAZ2B PHD–BRD to both H3 and H3K14ac, confirming that poly(ADP-ribosyl)ation modulates BAZ2B histone-binding affinity *in vivo* (Fig. 5, *C* and *D*).

### The positively charged linker is poly(ADP-ribosyl)ated by PARP1

**Figure 6.**
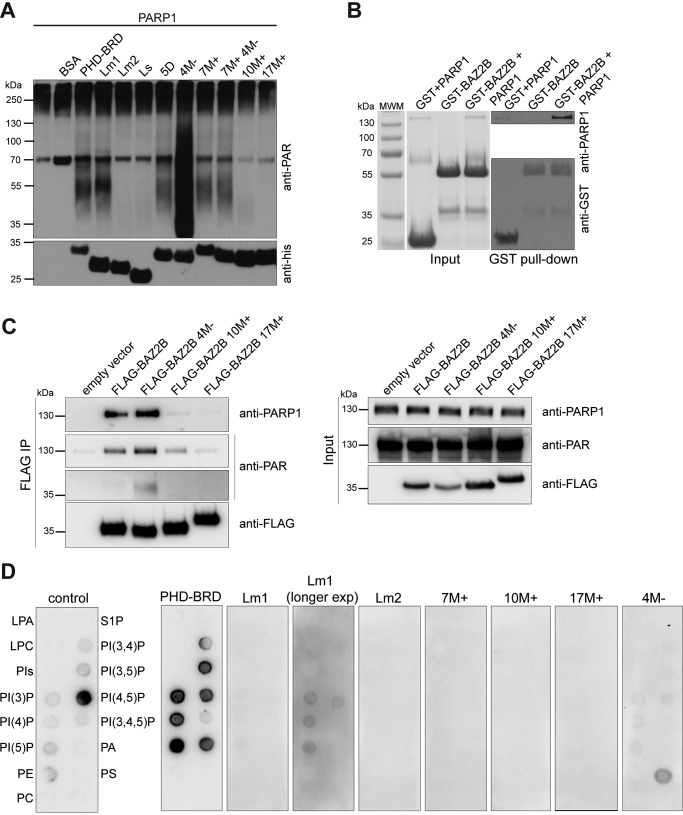
**Basic residues in the BAZ2B linker are poly(ADP-ribosyl)ated and bind phosphatidylinositol phosphates.** *A*, *in vitro* poly(ADP-ribosyl)ation assay of various BAZ2B constructs by activated PARP1. PARylation was detected by an anti-PAR antibody. *B*, *in vitro* GST pulldown assay shows binding of PARP1 to GST-BAZ2B PHD–BRD. 100% input and 50% eluate were loaded on the gel. Eluted proteins were detected by Western blotting. *C*, co-immunoprecipitation assay shows binding of PARP1 to FLAG-BAZ2B PHD–BRD. 10% input and 50% eluate were loaded on the gel. *D*, PIP strip assay showing binding of the control protein (*left panel)* and BAZ2B PHD-BRD wild-type (*right panel*). BAZ2B PHD–BRD mutants show reduced or no binding or an altered binding pattern.

Local sequence concentration of positively charged residues is often prone to poly(ADP-ribosyl)ation (PARylation) (28). An *in vitro* PARylation assay indeed showed that BAZ2B PHD–BRD is poly(ADP-ribosyl)ated, as judged by the smear of higher molecular weight (∼55 kDa) relative to the His_6_-BAZ2B PHD–BRD size (∼30 kDa) detected by an anti-PAR antibody (Fig. 6*A*). Constructs that lack positively charged residues, such as 10M^+^ (lysine-to-alanine mutations in the PHD-proximal part of the linker), 17M^+^ (lysine-to-alanine mutations in the PHD- and BRD-proximal part of the linker), and Lm2 (lacking the PHD-proximal part of the linker), were not PARylated (Fig. 6*A*). Surprisingly, alanine mutations of negatively charged aspartates and glutamates downstream from the PARylated motif (4M^−^) dramatically increased BAZ2B PARylation (Fig. 6*A*). This suggests that negatively charged residues form salt bridges with lysines and make them less accessible to modification. Furthermore, we confirmed the interaction between BAZ2B PHD–BRD and PARP1 in an *in vitro* GST pulldown assay (Fig. 6*B*) and an *in vivo* FLAG co-immunoprecipitation assay (Fig. 6*C*). In accordance with the *in vitro* PARylation assay, 10M^+^ and 17M^+^ mutants showed weak or no interaction with PARP1, respectively (Fig. 6*C*). The 4M^−^ mutant was PARylated *in vivo* and showed strong interaction with PARP1 (Fig. 6*C*).

### The BAZ2B linker binds phosphatidylinositol phosphates in vitro

Nuclear PIP-binding proteins share a common motif K/R-(X)_3–7_-K-X-K/R-K/R (29) also found in the BAZ2B linker within 10M+ and 7M+ stretches of basic residues (Fig. 4*B*). Therefore, we tested whether BAZ2B PHD-BRD binds different phosphoinositides using commercially available PIP strips (Fig. 6*D*). BAZ2B PHD–BRD was found to bind phosphatidylinositol 5-phosphate with strongest affinity, followed by PI3P, PI4P, PI(3,5)P, PI(4,5)P, and phosphatidic acid (Fig. 6*D*). Phosphoinositide binding was strongly reduced in the PHD–BRD Lm1 truncation lacking the BRD-proximal part of the linker, whereas the construct lacking the PHD-proximal part of the linker (Lm2) as well as 10M^+^ and 17M^+^ mutants did not show any binding (Fig. 6*D*). Interestingly, mutation of acidic residues in the linker (4M^−^) showed preferential binding to phosphatidylserine (Fig. 6*D*).

## Discussion

**Figure 7.**
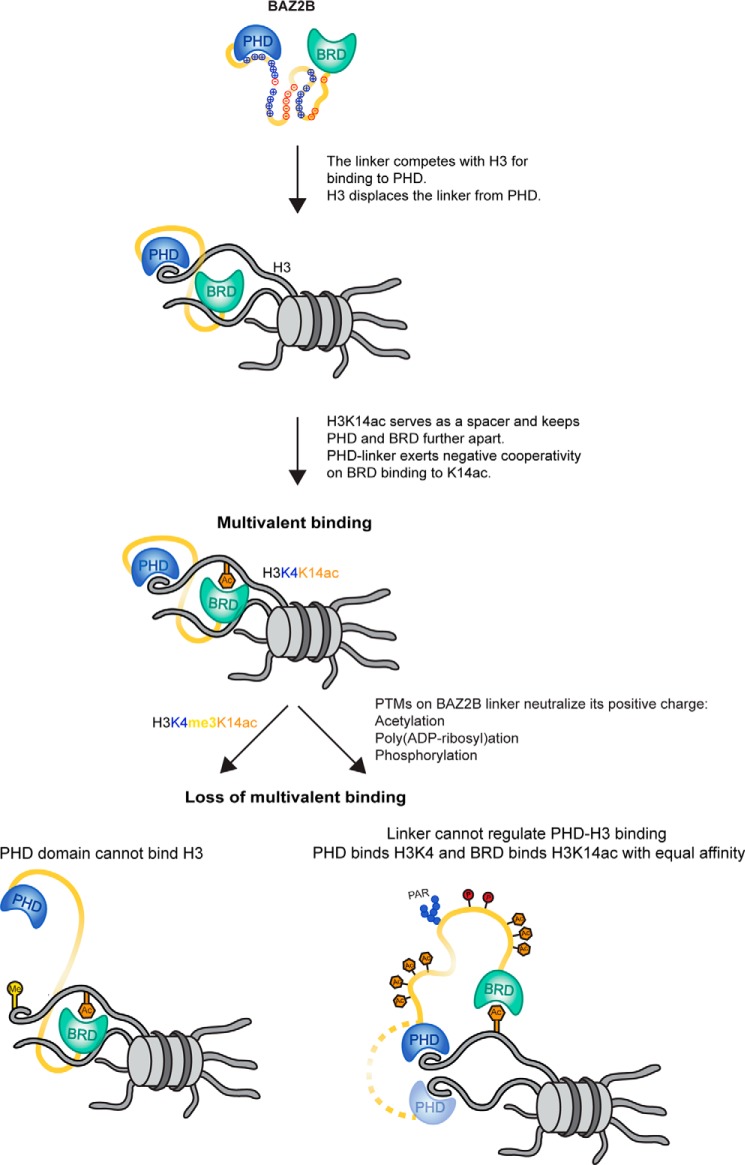
**A model for multivalent binding of BAZ2B PHD–BRD to histone H3K14ac.** The histone-mimicking interdomain linker competes with histone H3 for binding to the PHD and reduces the affinity of PHD–BRD toward H3K14ac. In *cis* multivalent binding of PHD–BRD to H3K14ac restricts the conformational freedom of the two domains. Methylation of H3K4 (*left*) or posttranslational modifications of the linker (*right*) leads to loss of multivalent binding. When H3K4 is methylated, the PHD cannot bind H3 and cannot exert negative cooperativity on BRD binding to K14ac. When the positive charges of the linker are neutralized by acetylation (*orange*) and negative charges are introduced by phosphorylation (*dark red*) or poly(ADP-ribosyl)ation (*blue*), the linker cannot keep the PHD in close proximity to the BRD, and the two domains act in an independent manner, binding the same H3 tail or two different H3 tails (*dotted linker line*).

Multivalent chromatin engagement is an important aspect of chromatin-associated protein functions (13, 30, 31). At least 37 known human proteins have the capacity for a multivalent readout via two or more PTM-binding modules (reader domains). BAZ2B is a multivalent histone reader of unknown biological function. The recent structural data revealed that the C terminus of BAZ2B harbors a PHD zinc finger that is involved in binding unmodified H3 and a BRD that binds specifically H3K14ac (7). Both domains are tethered via a 72-residue-long linker that is inherently unstructured. In this study, we analyzed the structural determinants of PHD-H3 binding, the mode of cooperation between the BAZ2B PHD and BRD, and the effect of the histone-mimicking linker on histone binding. We showed that two aspartates in the PHD (Asp^1950^ and Asp^1953^) are crucial for binding histone H3 at position Arg^2^, that PHD–BRD binds H3K14ac in a polyvalent manner, that the PHD linker domain negatively modulates binding of H3K14ac by the BRD, and that the interdomain linker competitively modulates H3 binding to the PHD through transient interactions with the same binding site (Fig. 7). This modulation of binding is lost when the PHD cannot bind H3 because of H3 methylation at Lys^4^ or when the linker cannot compete with H3 for PHD binding due to neutralization of its positive charge (Fig. 7).

Reader domains are independently folded domains that are often linked by intrinsically disordered regions (32). IDR flexibility and amino acid composition may serve different purposes. IDR flexibility enables spatial search of the domains for their cognate substrates through so-called “fly-casting” mechanisms, whereas IDR composition can modulate substrate-binding affinity and protein activity, facilitate regulation by providing sites for posttranslational modifications, and facilitate the assembly of membrane-less organelles as a hub for integration of diverse signaling pathways (17). Does the intrinsically disordered BAZ2B linker serve a purpose? We found that the PHD-proximal part of the BAZ2B linker reduces the affinity of PHD–BRD toward H3 almost 10-fold compared with the PHD alone and that it can specifically interact with the PHD domain in *trans*. The linker thus competes with H3 for binding to the same site in the PHD domain.

How does the linker compete with H3 for binding to the PHD? The PHD-proximal part of the linker resembles histone H3 and is rich in basic residues (mainly lysines) that are highly clustered, as observed previously for IDRs (33). Alanine mutations of positively charged residues within the PHD-proximal part of the linker (10M^+^) increased the H3-binding affinity of PHD–BRD to the level of the PHD alone, indicating that these linker residues modulate H3 binding to the PHD domain. This suggests that the PHD-proximal part of the linker directly modulates the binding of the PHD domain to H3 and, as a consequence, determines the overall affinity of PHD–BRD toward the acetylated histone substrate due to the polyvalent binding mode of the PHD–BRD.

Does BAZ2B PHD–BRD bind H3K14ac in *cis* or in *trans*? A previous study concluded that BAZ2B PHD–BRD binds H3K14ac in *trans* (23). The authors based their conclusions on a SAXS experiment that indicated a more extended conformation of the complex of tandem domains BAZ2B PHD–BRD upon binding H3K14ac (23). Our data, however, support an in *cis* binding mode: PHD–BRD tandem domains bind H3 or H3K14ac with reduced affinity compared with single PHD or BRD, respectively; PHD–BRD binds H3(1–21aa)K14ac with lower affinity compared with H3(11–21aa)K14ac; and PHD–BRD binds H3K4me3K14ac with higher affinity comparable with the affinity of BRD for K14ac. Moreover, our paramagnetic relaxation enhancement data explain the observed increase in radius of gyration in SAXS measurements by showing that the separation between the PHD and BRD increases due to bivalent H3K14ac binding. Although our data point to an in *cis* binding mode as more favorable, we cannot exclude an in *trans* binding mode.

How does the BAZ2B linker compare with H3? The resemblance of the PHD-proximal part of the BAZ2B linker to the histone substrate represents an example of intramolecular mimicry. Although molecular mimicry conventionally refers to a phenomenon in immune biology where pathogens mimic host proteins to disrupt host functions, it can also cover a broader concept where a protein (or protein domain) binds a target that is structurally similar to its cognate substrate (34). A recent example of intramolecular mimicry comes from the p53 inhibitor MDMX (murine double minute X), where the N-terminal p53-binding pocket can also bind a peptide within its central disordered regions in a structurally analogous manner to p53, resulting in MDMX autoinhibition (35). Histone mimicry has been described for the histone methyltransferase G9a, which competes for the binding to the histone-bound heterochromatin protein 1 (HP1) (36), the transcriptional factor SNAIL1, which binds histone demethylase LSD1-CoREST (lysine-specific demethylase 1–co-repressor RE1-silencing transcription factor) by mimicking H3 (37), and the NS1 (non-structural protein 1) protein of the influenza virus, which binds the transcription elongation factor hPAF1C (human RNA polymerase II-associated factor 1 complex) and the chromatin remodeling factor CHD1 (chromodomain helicase DNA-binding protein 1) by mimicking H3 (38, 39). Notwithstanding the high degree of similarity between the BAZ2B PHD-proximal linker and H3, replacement of H3R2 with lysine residues in the BAZ2B linker prevents intercalation mediated by the planar hydrophobic moiety of the guanidinium group of arginine.

How are the physicochemical properties of the BAZ2B linker regulated? Intrinsically disordered regions are enriched for sites that can be posttranslationally modified, such as lysine and serine, and are highly accessible to the modifying enzymes (40). Most of the known phosphorylation sites reside within IDRs (41). Ser^2014^ and Thr^2019^ in the BAZ2B linker region were already found to be phosphorylated during embryogenesis (42), whereas the abundant lysine residues are likely prone to *in vivo* acetylation or poly(ADP-ribosyl)ation, which would equally neutralize the linker positive net charge. Linker PTMs can change structural properties of the linker, fine-tune linker-mediated interactions by altering its physicochemical properties, and facilitate protein–protein interactions, as they can be recognized more easily by the cognate readers due to higher flexibility and accessibility of the linker region (40).

How can PTMs affect structural properties of the linker and fine-tune linker-mediated interactions? Although unstructured as independent entities, IDRs may fold upon binding their substrates (“disorder-to-order transition”) or because of electrostatic changes induced by PTMs (40, 43). PTMs may (de)stabilize local secondary structure or even induce global conformational changes of IDRs (40). PTMs neutralizing positively charged residues in the BAZ2B linker, such as acetylation, PARylation, or phosphorylation, could change the conformation of the linker and the relative positioning of the two domains as a result (Fig. 7). Diverse PTM combinations on the linker may generate interconvertible linker conformations that would support a spectrum of histone-binding affinities. PTMs neutralizing the linker positive charge may abrogate the inhibitory effect of the linker on PHD–H3 binding and the multivalent binding of PHD–BRD to H3K14ac (Fig. 7). Although we showed that reduction of cellular PAR levels weakens the binding of BAZ2B PHD–BRD to histone peptides, a comprehensive analysis of PTM-mediated regulation of the BAZ2B linker will be the subject of future studies.

In addition to covalent adjustments of the physicochemical properties of the linker, non-covalent binding of the phosphatidylinositol phosphate ligand to the positively charged residues may abrogate the inhibitory effect of the linker on PHD–H3 interactions. PIP5 was already shown to alleviate inhibition of the polybasic linker in UHRF1 on tandem Tudor domain–H3K9me3 binding (20) and to promote binding of ING2 (inhibitor of growth protein 2) to its target promoters (44).

Can the linker mediate BAZ2B assembly into macromolecular complexes? IDRs are frequently involved in protein–protein interactions that govern transcription, cell cycle regulation, or cell signaling (17). IDRs may also undergo intracellular liquid–liquid phase separation into membrane-less organelles, which is often promoted by PTMs (17). Given its modification by PARylation and its interaction with PARP1, the BAZ2B linker may promote BAZ2B assembly into liquid droplets seeded by PARylation (45). Alternatively, or additionally, the BAZ2B linker may mediate its targeted enrichment within nucleoli, a membrane-less compartment containing rRNA and rRNA-binding proteins, where its close homolog BAZ2A was found to bind to silent rRNA genes after passage of the replication fork and recruit chromatin modifiers to re-establish silent chromatin (21).

Collectively, we reveal an example of intramolecular mimicry mediated by an intrinsically disordered linker region of the multivalent histone binder BAZ2B, which negatively regulates histone H3 binding to BAZ2B PHD by acting as a histone mimic. We show that the modulation of PHD-H3 binding by the histone-mimicking linker negatively affects in *cis* binding of the PHD–BRD tandem domains to H3K14ac. We pin down the negative effect of the linker to its positively charged cluster, which competes with H3 for the binding to the PHD. Neutralization of the linker positive charge by posttranslational modifications such as acetylation, PARylation, or phosphorylation may represent a cellular regulatory mechanism to increase the binding affinity of BAZ2B to its histone substrate. Autoinhibition by intramolecular mimicry and its release by posttranslational modifications may therefore represent a more common mechanism to regulate the dynamics of protein interactions than envisaged previously.

## Experimental procedures

### Protein expression and purification

For N-terminal His_6_ tagging, BAZ2B constructs were cloned into the pETM11 vector. For N-terminal GST tagging, BAZ2B PHD–BRD was cloned into pGEX-4T1. Site-directed mutagenesis was performed with Phusion polymerase (46) according to the FastCloning protocol (47). Proteins were expressed in *Escherichia coli* Rosetta2 (DE3) strain (Novagen). Expression was induced with 0.5 mm isopropyl 1-thio-β-d-galactopyranoside (Sigma) in the presence of 0.1 mm ZnCl_2_ (3 h, 30 °C), followed by sonication in binding buffer (500 mm NaCl, 25 mm Tris-Cl (pH 7.4), and 20 mm imidazole) in the presence of 0.1% Nonidet P-40 and protease inhibitors. Proteins were purified using HisTrap nickel resin (Thermo Fisher Scientific), eluted with elution buffer (500 mm NaCl, 25 mm Tris-Cl (pH 7.4), and 500 mm imidazole), and dialyzed overnight against 50 mm NaCl and 25 mm Tris-HCl (pH 7.4). For the GST purification, cells were lysed by sonication in 150 mm NaCl, 50 mm Tris-Cl (pH 7.4), protease inhibitors, and 0.1% Nonidet P-40. Following incubation with glutathione-agarose beads (Thermo Fisher Scientific), proteins were eluted with 20 mm glutathione, 50 mm Tris (pH 7.4), and 150 mm NaCl.

### ^15^N and ^13^C labeling

Transformed Rosetta2 cells were grown in M9 minimal medium containing ^15^N-labeled ammonium chloride (Eurisotop) without or with ^13^C-labeled glucose (Eurisotop). Protein expression and purification were performed as described above. Purified proteins were dialyzed overnight against 100 mm NaCl in 10 mm sodium phosphate buffer (pH 6.8).

### Peptide synthesis

Histone peptides were synthesized and purified as described previously (48) and provided by the IMP Peptide Synthesis Facility.

### ITC

All calorimetric measurements were carried out at 25 °C using an iTC200 microcalorimeter (GE Healthcare) with a 200-μl cell capacity and a 40-μl syringe volume. The initial injection of 0.4 μl was followed by 19 2-μl injections with a spacing time of 150 s. In some cases, a different experimental setup with 10 injections of 1 μl followed by 14 injections of 2 μl was required to gain a more sigmoidal titration curve. Concentrations ranged from 150–260 μm for the sample and 1.5–4 mm for the titrant. All experiments were carried out at *T* = 298.15 K and a stirring speed of 800 rpm. Control injections were subtracted from sample measurements to correct for heat development during dilution. MicroCal Origin 7.0 software was used for analysis; the data points were fitted according to a one-site binding model. The summary of raw data can be found in supplemental Table 1.

### Paramagnetic relaxation enhancement

Thr^6^ of the N-terminal H3(1–21) peptide was replaced by a Cys and subsequently tagged with the nitroxide spin label *S*-(1-oxyl-2,2,5,5-tetramethyl-Δ3-pyrroline-3)methyl methanethiosulfonate (MTSL, Santa Cruz Biotechnology). The peptide was subsequently separated from DTT by a PD-10 desalting column (GE Healthcare). Absorbance was measured at 412 nm, and a 2.5-fold excess of MTSL was added, followed by incubation at 37 °C for 3 h. The measurements were recorded on a Bruker AV600HD+ at 25 °C. The intensity ratio of the oxidized and reduced (50 mm ascorbic acid) BAZ2B(PHD-BRD)–H3(1–21) complex was measured. Ratios of cross-peaks between oxidized and reduced states were calculated (Iox/Ired) and depicted as a temperature coding using home-written routines based on NMRPipe (49).

### NMR spectroscopy

Except for the MTSL measurements, all NMR spectra were recorded at 25 °C on Varian or Bruker spectrometers operating at 500, 600, and 800 MHz. 10% D_2_O was used as a lock solvent and added to the BAZ2B H3(K14ac) complexes dissolved in 10 mm sodium phosphate buffer (pH 6.8) and 100 mm NaCl. Protein concentrations were typically between 0.3–1.6 mm with a 1:1 protein:peptide stoichiometry. All NMR datasets were processed using NMRPipe. For resonance assignment, triple resonance CBCA(CO)NH, HNCACB, HNCA, and HN(CO)CA experiments were performed (50). The assignments were done using Sparky software. Peak intensities were normalized by assigning M1927 as the most intense peak. Chemical shift perturbations were calculated from the combined amide ^1^H^N^ and ^15^N shift changes as shown in Equation 1.

(Eq. 1) $$\Delta \delta =\sqrt{{\left(5\Delta \delta^{HN}\right)}^{2}+{\left(\Delta \delta^{N}\right)}^{2}}$$

### Antibodies

The following antibodies were used for Western blotting: anti-FLAG M2-peroxidase clone M2 (1:10,000, Sigma), rabbit anti-PAR (1:1000, Trevigen), rabbit anti-PARP1 (1:1000, Cell Signaling Technology), mouse anti-His (1:5000, GE Healthcare), and mouse anti-GST (1:500, a gift from Egon Ogris). Secondary HRP-conjugated antibodies for Western blotting (Jackson ImmunoResearch Laboratories) were used at 1:10,000 dilution.

### In vitro poly(ADP-ribosyl)ation assay

2 units of PARP1 (Trevigen, 4668-500-01) were incubated with 1.5 μg of BAZ2B proteins in the presence of 200 μm NAD (Trevigen), activated DNA (Trevigen), 50 mm Tris (pH 7.5), and 50 mm NaCl. The reactions were analyzed by Western blotting with anti-PAR antibody.

### GST pulldown

10 μg of GST or GST-BAZ2B PHD–BRD was incubated with 5 units of PARP1 (Trevigen, 4668-500-01) in 250 μl of pulldown buffer (20 mm Tris (pH 7.5), 100 mm NaCl, 0.05% Triton, and protease inhibitors) for 2 h with rotation at 4 °C. 20 μl of glutathione-agarose (Thermo Fisher Scientific) was added and incubated for an additional 2 h. The beads were washed four times with 300 μl of pulldown buffer and eluted by boiling in SDS sample buffer.

### Co-immunoprecipitation

BAZ2B PHD–BRD was cloned into pDONR221 and transferred into pDEST N3xFLAG by Gateway cloning. 8 μg of FLAG-BAZ2B PHD–BRD was transfected into a 10-cm dish containing HEK293T cells using PEI (Polysciences). HEK293T cells were cultured in DMEM (Sigma, 4.5 g/liter of glucose) supplemented with 10% fetal bovine serum (Sigma), 1% l-glutamine (Sigma), and 1% penicillin-streptomycin (Sigma) under 5% CO_2_ at 37 °C. 48 h after transfection, the cells were harvested and lysed in 50 mm Tris-Cl (pH 8), 150 mm NaCl, 1% Triton, 1 mm DTT, 50 units/ml benzonase (Novagen), and protease inhibitors (EDTA-free, Roche). Anti-FLAG M2 magnetic beads (Sigma) were equilibrated by washing the beads twice with TBS. Lysates were incubated with the beads for 2 h at 4 °C with rotation. The beads were washed three times with lysis buffer and eluted with 3× FLAG peptide (Sigma).

### Histone peptide pulldown

100 μg of biotinylated histone peptides (synthesized by Mathias Madalinski, IMP) was coupled to 50 μl of Dynabeads MyOne Streptavidin C1 (Thermo Fisher Scientific). HEK293T cells were transfected with FLAG-BAZ2B PHD–BRD, and nuclear extracts were prepared as described in Ref. 51. 1 mg of nuclear extract was incubated with beads coupled to the biotinylated peptides or empty beads for 2 h at 4 °C. The beads were washed six times with 500 mm NaCl, 20 mm Hepes (pH 7.9), 10% glycerol, 1% Triton, and 1 mm DTT. Elution was performed at 25 °C in 25 μl of sample buffer by shaking at 1400 rpm for 30 min.

### PIP strips

PIP strips (Echelon) were blocked in 3% BSA for 1 h. The membrane was incubated with 0.5 μg/ml of the PI(4,5)P-specific binding protein (GST-tagged G-4501, positive control) or His_6_-tagged BAZ2B PHD–BRD wild-type and mutant constructs for 1 h at room temperature. The membranes were washed three times and incubated for 1 h at room temperature with anti-GST as a positive control or anti-His for BAZ2B. Following three washing steps, the membranes were incubated with anti-mouse-HRP for 1 h at room temperature and washed three times before chemiluminescent detection.

## Author contributions

S. K. designed experiments, cloned and purified His6-tagged BAZ2B constructs, performed ITC measurements and analysis, assigned the residues of the BAZ2B BRD, and wrote the manuscript. G. K. designed experiments, performed NMR measurements and analysis, and assigned the BAZ2B PHD. T. K. purified the proteins used in the histone binding screen. E. S. performed and S. K. supervised the histone binding screen. R. K. designed the NMR experiments. D. S. designed the study; performed *in vivo* histone binding, GST pulldown, co-immunoprecipitation, PARylation, and PIP strip assays; purified proteins; analyzed the data; and wrote the manuscript. All authors approved the final version of the manuscript.

## Supplementary Material

Supplemental Data

## Footnotes

3 The abbreviations used are:

PTM: posttranslational modification
BRD: bromodomain(s)
PHD: plant homeodomain(s)
IDR: intrinsically disordered region
H3K14ac: H3 acetylated at Lys^14^
H3K9me3: H3 trimethylated at Lys^9^
ITC: isothermal titration calorimetry
aa: amino acids
HSQC: heteronuclear single quantum coherence
SAXS: small angle X-ray scattering
MTSL: *S*-(1-oxyl-2,2,5,5-tetramethyl-Δ3-pyrroline-3)methyl methanethiosulfonate
UHRF1: ubiquitin-like PHD RING finger 1
PAR: poly(ADP-ribosyl)
PARylation: poly(ADP-ribosyl)ation
PARP: poly(ADP-ribose) polymerase
PIP: phosphatidylinositol phosphate
PI3P: phosphatidylinositol (3) phosphate
PI4P: phosphatidylinositol (4) phosphate
PI(4,5)P: phosphatidylinositol (4,5) bisphosphate.
