# Supplementary material for: A histone-mimicking interdomain linker in a multidomain protein modulates multivalent histone binding
Source: J Biol Chem. 2017 Sep 1;292(43):17643–57. doi: 10.1074/jbc.M117.801464 (PMC5663869; doi:10.1074/jbc.M117.801464)
Supplement: Supplemental Data [file supp_292_43_17643__index.html]

A histone-mimicking interdomain linker in a multi-domain protein modulates multivalent histone binding — A histone-mimicking interdomain linker in a multidomain protein modulates multivalent histone binding — BAZ2B interdomain linker modulates H3 binding — Supplemental Data 

# A histone-mimicking interdomain linker in a multidomain protein modulates multivalent histone binding

## Supplemental Data

- Supplemental Material (.pdf, 4.2 MB) - Supplemental Material
