# Supplementary material for: A histone-mimicking interdomain linker in a multidomain protein modulates multivalent histone binding
Source: J Biol Chem. 2017 Sep 1;292(43):17643–57. doi: 10.1074/jbc.M117.801464 (PMC5663869; doi:10.1074/jbc.M117.801464)

Supplemental Information to

A histone-mimicking interdomain linker in a multi-domain protein modulates multivalent histone binding

Kostrhon et al.

Supplemental Results

A high-throughput histone peptide binding screen reveals specificity of BAZ2B PHD-BRD towards H3K14ac—We performed a high-throughput DELFIA screen using a library of biotinylated histone peptides with various combinations of modifications. Biotinylated histone peptides were prebound to streptavidin-coated DELFIA plates and incubated with His_6-tagged BAZ2B PHD, BRD or PHD-BRD domains (Supplemental Fig. 8). DELFIA screen performed with BAZ2B domains showed reproducibly strong binding to H3K14ac, which is reduced when H3K4 is methylated (Supplemental Fig. 8).

Supplemental Experimental Procedures

Histone peptide binding screen–96 biotin-labelled histone peptides unmodified or bearing different modifications (acetylation, phosphorylation, methylation) were used in the screen. 20µl peptide solution was aspirated from the 96-Deepwell plate and dispensed to 384-well DELFIA streptavidin-coated white plates (Perkin Elmer) in quadruplicates using the pipetting robot JANUS. After spinning down plates and incubating them at room temperature for 1 hour, 20µl of purified His_6-tagged BAZ2B PHD, BRD or PHD-BRD was added in technical duplicates in the FI Buffer (50 mM Tris pH7.5, 150 mM NaCl, 0.05% Tween-40, 25mM DTPA, 0.2% BSA, 0.05% bovine γ-globulins) and incubated for 3 hours at room temperature. Unbound peptides and proteins were removed by washing the plates 3 times with 100µl 1x WASH buffer II (50mM Tris-Cl pH7.5, 150mM NaCl, 0.05% Tween-20). 50µl of 1µg/ml anti-His.H8 (Millipore) and 200ng/ml DELFIA-Eu-N1-anti-mouse (Perkin Elmer) antibody in FI buffer was added to 384-well DELFIA plates via Multidrop Dispenser. Subsequently, the plates were centrifuged and incubated at room temperature for 1 hour. Unbound antibodies were removed by washing the plate 3 times with 100µl 1x WASH buffer II. 50µl enhancement solution (PerkinElmer) was transferred to each plate via Multidrop Dispenser. After incubating the plates 45 minutes at room temperature, Europium emission was measured via Envision. Data were analyzed using Pipeline Pilot 8.0. For data visualization TIBCO Spotfire 4.0.2 was used. The screen was performed twice with high reproducibility.

Supplemental Table 1. Summary of ITC data.

Histone peptide name	Histone peptide sequence	BAZ2B construct name	K_d (μM)	*N*	ΔH (kcal/mol)	ΔS (cal/mol/deg)
H3 (1-21)	*ARTKQTARKSTGGKAPRKQLA*	PHD-BRD WT	*54.30±6.68*	*1.18±0.16*	*-5.71±0.17*	*0.38±0.82*
		PHD-BRD R1938A	*51.29±0.79*	*0.35±0.02*	*-5.17±0.04*	*2.31±0.18*
		PHD-BRD K1939A	*46.32±2.46*	*0.41±0.02*	*-5.76±0.03*	*0.52±0.00*
		PHD-BRD D1941A	*158.12±27.06*	*0.25±0.08*	*-17.87±7.07*	*-42.45±23.95*
		PHD-BRD N1942A	*32.95±0.27*	*0.98±0.02*	*-5.54±0.06*	*1.94±0.21*
		PHD-BRD E1943A	*86.64±14.17*	*0.93±0.03*	*-5.78±0.54*	*-0.74±2.14*
		PHD-BRD E1944A	*44.79±2.60*	*1.25±0.02*	*-5.18±0.04*	*2.52±0.23*
		PHD-BRD	*111.46±2.17*	*1.46±0.04*	*-6.29±0.22*	*-2.98±0.76*
		PHD-BRD D1950A	*1876.99±292.21*	*0.39±0.26*	*-12.83±7.82*	*-30.43±25.98*
		PHD-BRD D1953A	*130.19±24.37*	*1.18±0.02*	*-3.56±0.22*	*5.89±1.11*
		PHD-BRD Lm1	*80.30±14.94*	*0.86±0.02*	*-6.78±0.60*	*-4.22±2.66*
		PHD-BRD Lm2	*18.12±0.13*	*1.66±0.01*	*-5.10±0.01*	*4.62±0.04*
		PHD-BRD Ls	*51.98±2.96*	*1.04±0.03*	*-6.15±0.31*	*-1.01±1.16*
		PHD-BRD 7M+	*17.65±3.32*	*0.67±0.01*	*-5.08±0.29*	*4.77±1.35*
		PHD-BRD 4M-	*104.10±22.80*	*0.28±0.11*	*-9.16±2.92*	*-12.21±9.99*
		PHD-BRD 5D	*17.45±1.90*	*0.64±0.06*	*-5.84±0.26*	*2.19±1.08*
		PHD-BRD 7M+ 4M-	*59.93±1.79*	*0.82±0.02*	*-5.36±0.19*	*1.35±0.70*
		PHD-BRD 10M+	*5.74±1.45*	*0.34±0.01*	*-5.18±0.32*	*6.67±1.59*
		PHD-BRD 17M+	*8.97±0.12*	*0.68±0.12*	*-5.10±0.38*	*5.99±1.24*
		PHD WT	*5.78±0.43*	*0.61±0.01*	*-6.87±0.13*	*0.93±0.30*
		PHD R1938A	*7.77±0.36*	*0.31±0.03*	*-6.57±0.20*	*1.33±0.70*
		PHD K1939A	*6.31±0.40*	*1.01±0.02*	*-6.06±0.49*	*3.49±2.50*
		PHD D1941A	*51.19±10.54*	*0.05±0.04*	*-50.95±41.25*	*-151.20±138.80*
		PHD N1942A	*5.84±0.96*	*0.24±0.00*	*-9.32±0.02*	*-7.28±0.24*
		PHD E1943A	*13.38±1.57*	*0.71±0.00*	*-5.89±0.17*	*2.57±0.79*
		PHD E1944A	*6.21±0.08*	*0.73±0.03*	*-5.76±0.05*	*4.53±0.19*
		PHD E1943+1944A	*13.34±0.18*	*0.38±0.19*	*-6.95±0.31*	*-1.00±1.05*
		PHD D1950A	*474.20±11.24*	*0.33±0.21*	*-15.02±9.85*	*-35.15±32.96*
		PHD D1953A	*59.74±1.61*	*0.81±0.07*	*-3.78±0.15*	*6.67±0.57*
		PHD D1950+53A	*180.82±83.73*	*0.32±0.21*	*-1.69±1.29*	*11.69±5.32*
		PHD-Linker WT	*52.65±1.11*	*1.14±0.01*	*-5.58±0.26*	*0.87±0.93*
		PHD Lm	*23.33±1.30*	*0.54±0.01*	*-6.93±0.08*	*-2.04±0.36*
		PHD-Linker 7M+	*23.05±0.93*	*1.09±0.01*	*-4.50±0.03*	*6.12±0.18*
		PHD-Linker 4M-	*110.93±0.92*	*0.92±0.05*	*-5.59±0.06*	*-0.63±0.23*
		PHD-Linker 5D	*22.33±1.14*	*0.50±0.01*	*-5.42±0.04*	*3.13±0.25*
		PHD-Linker 7M+	*70.43±1.14*	*0.99±0.03*	*-5.50±0.10*	*0.54±0.36*
		PHD-Linker 10M+	*5.46±1.17*	*0.75±0.01*	*-6.17±0.00*	*3.45±0.44*
		PHD-Linker 17M+	*3.71±0.10*	*1.01±0.06*	*-5.02±0.29*	*7.98±1.04*
H3K14ac (1-21)	*ARTKQTARKSTGGKacAPRKQLA*	PHD-BRD	*24.27±1.70*	*0.78±0.02*	*-15.86±1.12*	*-32.00±3.96*
		PHD-BRD Lm1	*29.40±4.27*	*0.70±0.01*	*-12.70±0.63*	*-2.40±21.8*
		PHD-BRD Lm2	*25.46±0.58*	*1.45±0.01*	*-64.65±52.65*	*-18.70±0.50*
		PHD-BRD Ls	*40.33±0.49*	*0.80±0.01*	*-12.36±0.33*	*-21.35±1.15*
		PHD-BRD 7M+	*27.27±1.22*	*0.91±0.02*	*-11.96±0.10*	*-19.20±0.40*
		PHD-BRD 4M-	*24.15±1.63*	*0.85±0.01*	*-12.16±0.41*	*-19.60±1.50*
		PHD-BRD 5D	*29.61±6.88*	*0.50±0.09*	*-8.04±1.89*	*-6.18±6.82*
		PHD-BRD 7M+ 4M-	*35.38±1.25*	*0.55±0.03*	*-13.68±0.08*	*-24.60±1.10*
		PHD-BRD 10M+	*15.10±1.25*	*0.64±0.06*	*-12.92±0.62*	*-21.20±2.20*
		PHD-BRD 17M+	*17.14±1.49*	*0.94±0.07*	*-8.34±0.04*	*-6.16±0.04*
		BRD	*6.70±0.49*	*0.59±0.00*	*-9.53±0.04*	*-8.26±0.27*
		BRD Linker	*9.34±1.40*	*0.47±0.00*	*-7.26±0.54*	*-1.28±2.12*
		BRD Lm	*6.40±0.18*	*0.78±0.01*	*-7.31±0.07*	*-0.77±0.23*
		BRD Ls	*5.64±0.35*	*0.96±0.01*	*-7.12±0.06*	*0.16±0.09*
H3 (1-10)	*ARTKQTARKS*	PHD-BRD WT	*115.87±2.75*	*2.03±0.07*	*-4.07±0.15*	*4.28±0.64*
		PHD	*23.13±4.12*	*0.66±0.02*	*-4.53±0.19*	*6.08±0.42*
H3 (11-21)	*TGGKAPRKQLA*	PHD	*1959.76±1235.1*	*11.41±3.79*	*-0.85±0.20*	*10.06±2.14*
H3K14ac (11-21)	*TGGKacAPRKQLA*	PHD-BRD	*10.03±3.97*	*0.70±0.00*	*-4.28±0.45*	*8.68±2.32*
		BRD	*8.24±3.39*	*0.58±0.01*	*-5.32±0.93*	*5.61±3.99*

Histone peptide name	Histone peptide sequence	BAZ2B construct name	K_d (μM)	*N*	ΔH (kcal/mol)	ΔS (cal/mol/deg)
H3K4me1 (1-21)	*ARTKme1QTARKSTGGKAPRKQLA*	PHD-BRD	*88.40±5.06*	*1.94±0.18*	*-4.3±0.06*	*2.44±0.67*
		PHD	*10.22±0.60*	*0.82±0.02*	*-4.17±0.15*	*8.87±0.61*
H3K4me2 (1-21)	*ARTKme2QTARKSTGGKAPRKQLA*	PHD-BRD	*123.29±15.42*	*1.76±0.18*	*-2.92±0.44*	*8.11±1.72*
		PHD	*12.38±5.19*	*0.71±0.02*	*-4.42±0.10*	*6.90±0.30*
H3K4me3 (1-21)	*ARTKme3QTARKSTGGKacAPRKQLA*	PHD-BRD	*129.64±11.02*	*1.89±0.10*	*-1.71±0.18*	*12.05±0.75*
		PHD	*23.88±0.46*	*0.73±0.02*	*-3.71±0.21*	*8.56±0.82*
H3T6C (1-21)	*ARTKQCARKSTGGKAPRKQLA*	PHD-BRD	*148.45±33.37*	*1.59±0.03*	*-4.36±0.60*	*2.97±2.45*
		PHD	*33.22±3.81*	*0.74±0.02*	*-5.39±0.22*	*0.74±0.02*
H3K14ac T6C	*ARTKQCARKSTGGKacAPRKQLA*	PHD-BRD	*50.35±4.90*	*2.92±0.08*	*-6.16±0.01*	*-0.95±0.14*
		PHD	*26.99±1.02*	*4.86±0.14*	*-2614.50±0.05*	*26.99±1.02*
H3K4me1K14ac	*ARTKme1QTARKSTGGKacAPRKQLA*	PHD-BRD	*26.95±8.26*	*0.54±0.01*	*-9.41±4.86*	*-10.58±16.93*
H3K4me2K14ac	*ARTKme2QTARKSTGGKacAPRKQLA*	PHD-BRD	*18.91±2.41*	*0.45±0.02*	*-11.21±0.14*	*-15.95±0.75*
H3K4me3K14ac	*ARTKme3QTARKSTGGKacAPRKQLA*	PHD-BRD	*6.99±0.05*	*0.54±0.05*	*-7.43±1.07*	*-1.32±3.60*

Supplemental Figure Legends

SUPPLEMENTAL FIG. 1. **Mutation of negatively charged residues within the PHD finger abrogate the binding of BAZ2B PHD-BRD to histone H3**. (A) Differences in chemical shifts between BAZ2B PHD-BRD and PHD-BRD in complex with H3 visualized as color code. Major differences can be observed in the 3_{10} helix and the β2 strand. (B) Comparison of H3 binding affinities of BAZ2B PHD-BRD WT and various mutations within the PHD domain.

SUPPLEMENTAL FIG. 2. **^{15}N-HSQC spectra of the paramagnetic relaxation enhancement experiments**. (A) H3T6C binding to BAZ2B PHD-BRD. (B) H3T6C K14ac binding to BAZ2B PHD-BRD.

SUPPLEMENTAL FIG. 3. **Chemical shifts of the BAZ2B PHD domain within the PHD-BRD construct are induced by the linker**. Overlay of ^{15}N-HSQC spectra of BAZ2B PHD and PHD-BRD (A) or PHD-linker (B).

SUPPLEMENTAL FIG. 4. NMR titration of H3K14ac into BAZ2B PHD-BRD reveals auto-inhibitory effect of the linker on H3 binding to the PHD domain. (A) ^{15}N-HSQC spectra of BAZ2B PHD-BRD titration with H3K14ac. Colors indicate the following protein/peptide ratios: free (red), orange (1:0.5), 1:1 (yellow), 1:1.5 (pink), 1:2 (magenta), 1:4 (maroon). (B), (C) Chemical shift changes in BRD (B) and PHD (C) residues for different protein/peptide ratios. The data were fit with a single site binding model (B) and Hill function (C) to obtain estimated K_d values.

SUPPLEMENTAL FIG. 5. **The linker does not affect the binding of the BAZ2B BRD domain to H3K14ac.** Dissociation constants were determined by ITC.

SUPPLEMENTAL FIG. 6. **Conserved positively and negatively charged amino acids in the BAZ2B linker modulate H3 binding affinity**. (A) Conservation of positively charged amino acids in the BAZ2B linker sequence across different species. The sequences are: *Pan troglodytes* (K7DS26); *Homo sapiens* (Q9UIF8); *Xenopus tropicalis* (F6QSX8); *Gallus gallus* (Q9DE13); *Taeniopygia guttata* (H0ZPT1); *Mus musculus* (A2AUY4); *Myotis lucifugus* (G1NV17); *Cavia porcellus* (H0VSW1). (B) ITC measurement of dissociation constants for the binding of BAZ2B PHD-linker mutants to H3.

SUPPLEMENTAL FIG. 7. **PHD-proximal part of the linker affects the conformation of the BRD domain in the context of tandem domains.** Differences in chemical shifts between BAZ2B BRD and PHD-BRD (A) or PHD-BRD 10M+ (B) visualized with a color code.

SUPPLEMENTAL FIG. 8. **A high-throughput DELFIA screen reveals specificity of BAZ2B BRD and PHD-BRD towards H3K14ac**. (A) A heat map showing the strength of binding of His_6-tagged BAZ2B PHD, BRD and PHD-BRD to biotinylated histone peptides with different modifications (red = maximum binding; blue = minimum binding). (B), (C), (D) Scatter plots showing distribution of signal intensity for histone peptide binding of BAZ2B PHD (B), BRD (C) and PHD-BRD (D) domains.

Supplemental Figure 1

A

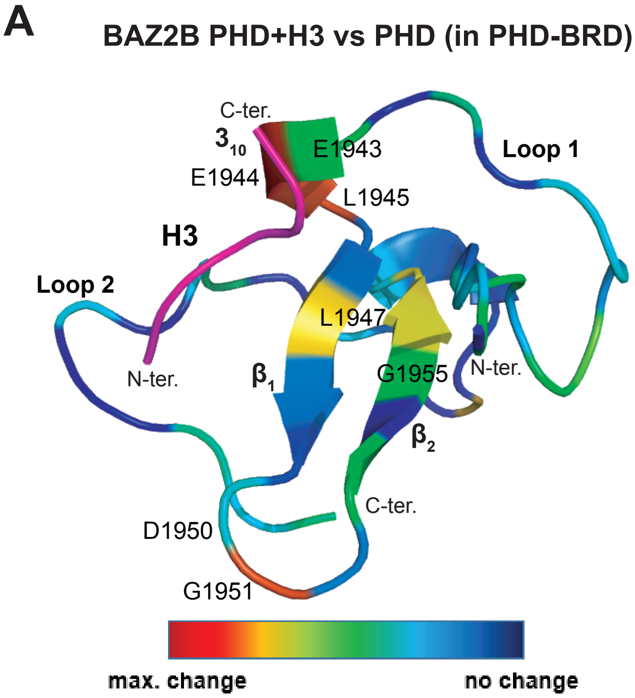

B

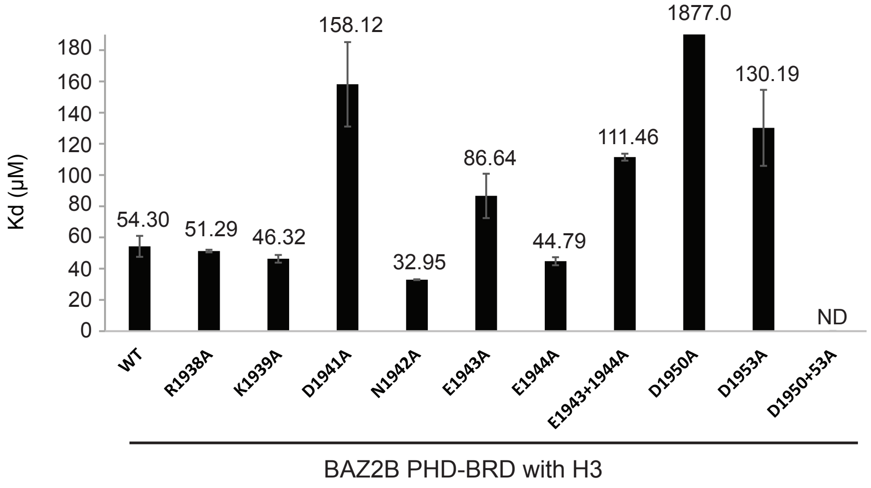

Supplemental Figure 2

A

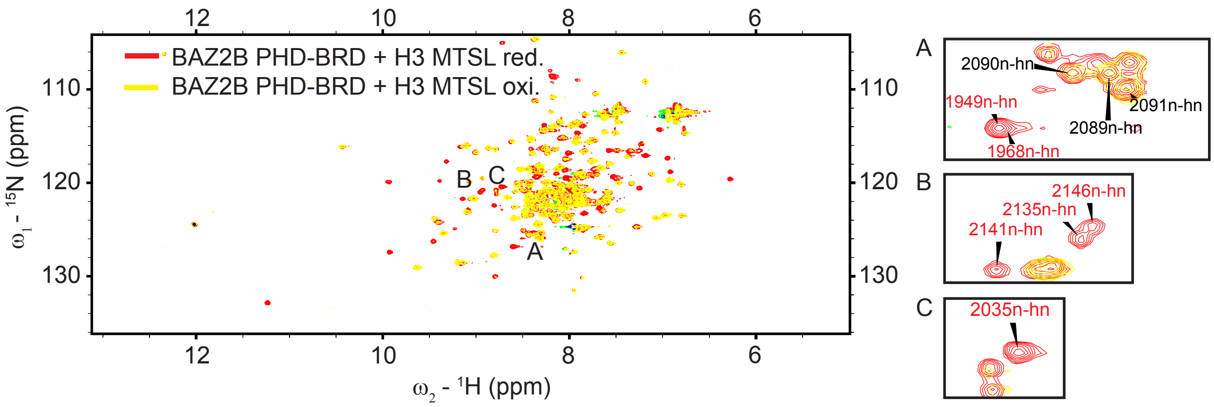

B

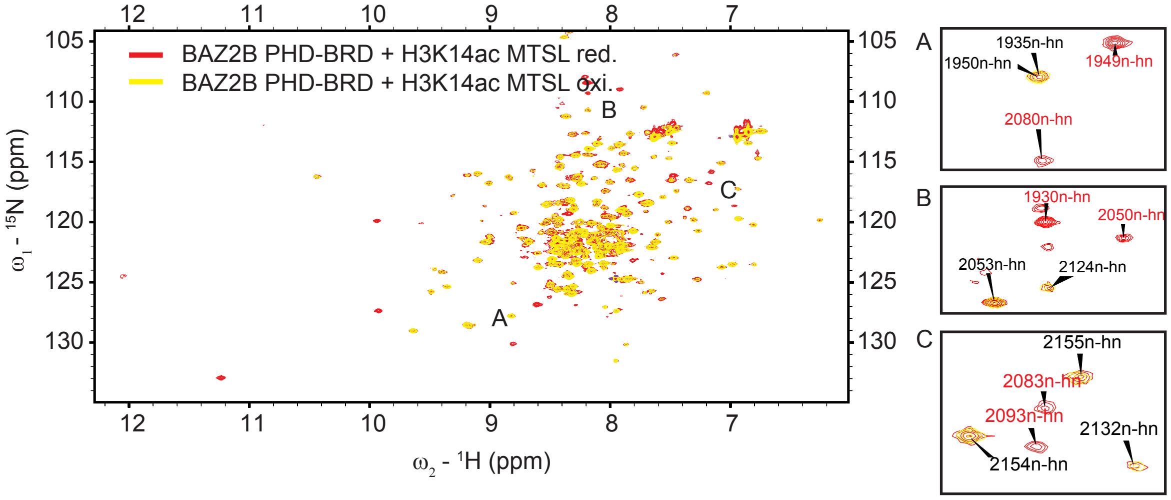

Supplemental Figure 3

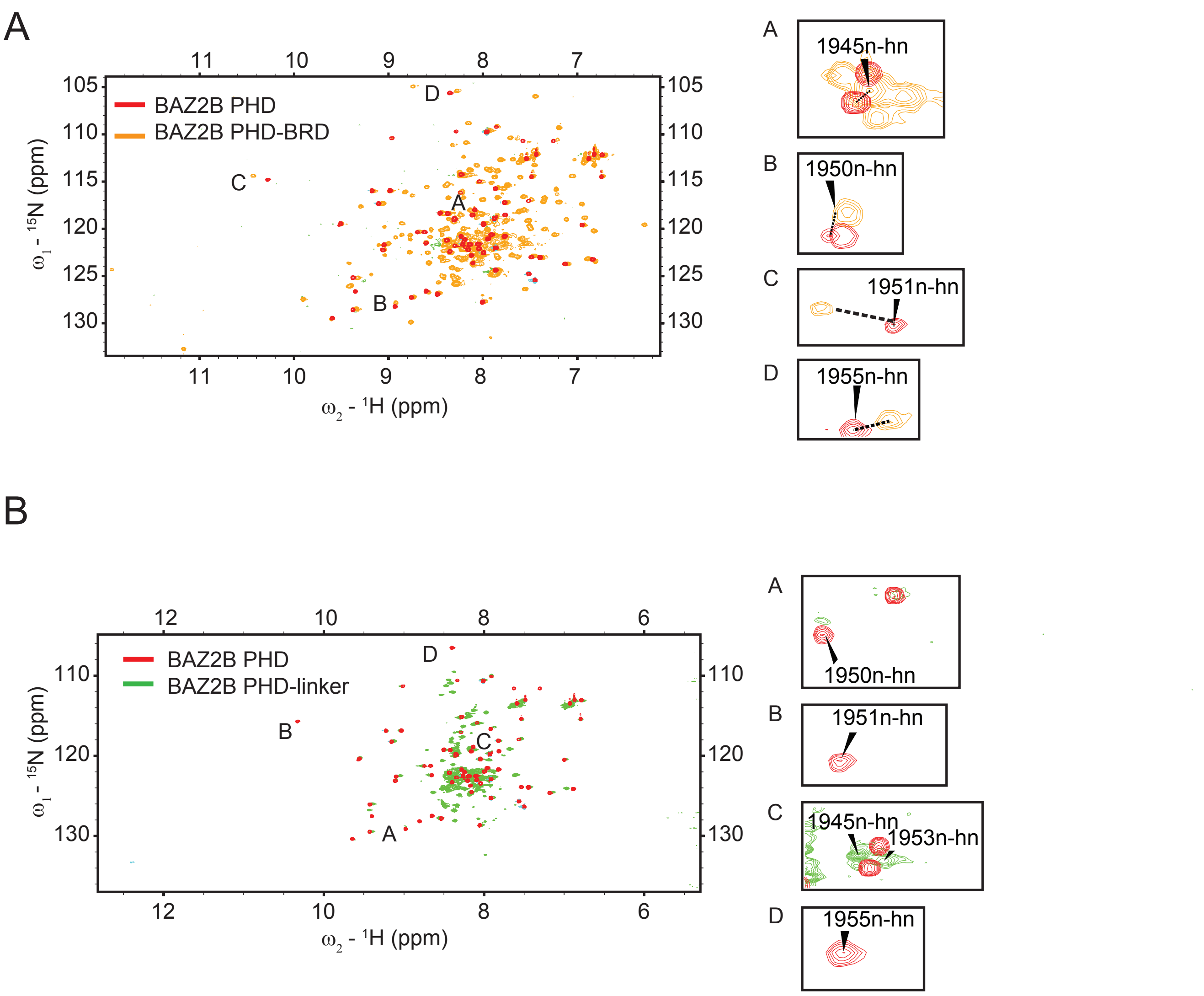

Supplemental Figure 4

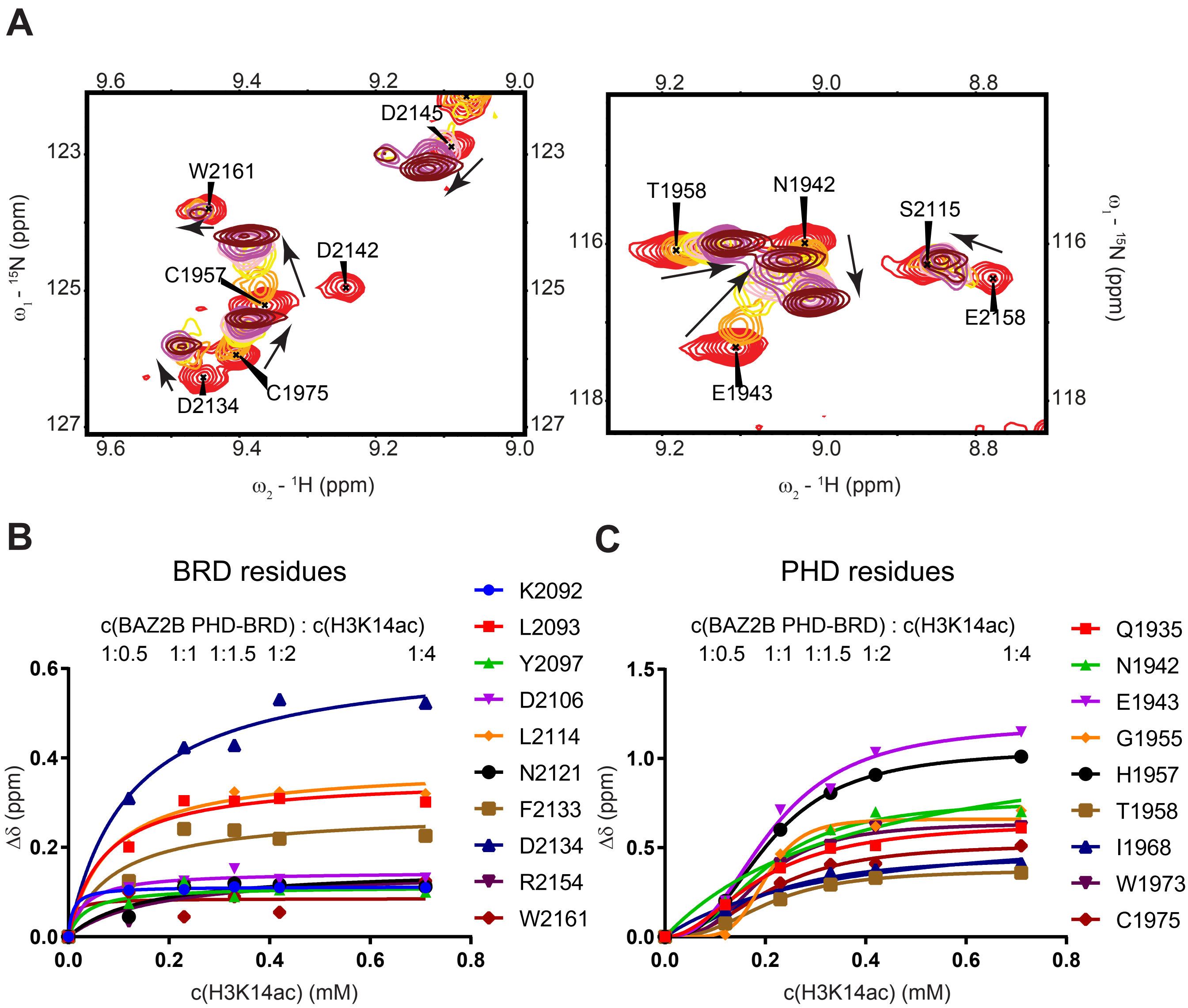

Supplemental Figure 5

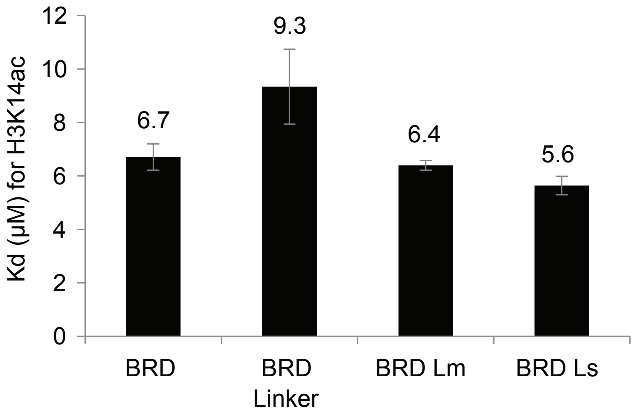

Supplemental Figure 6

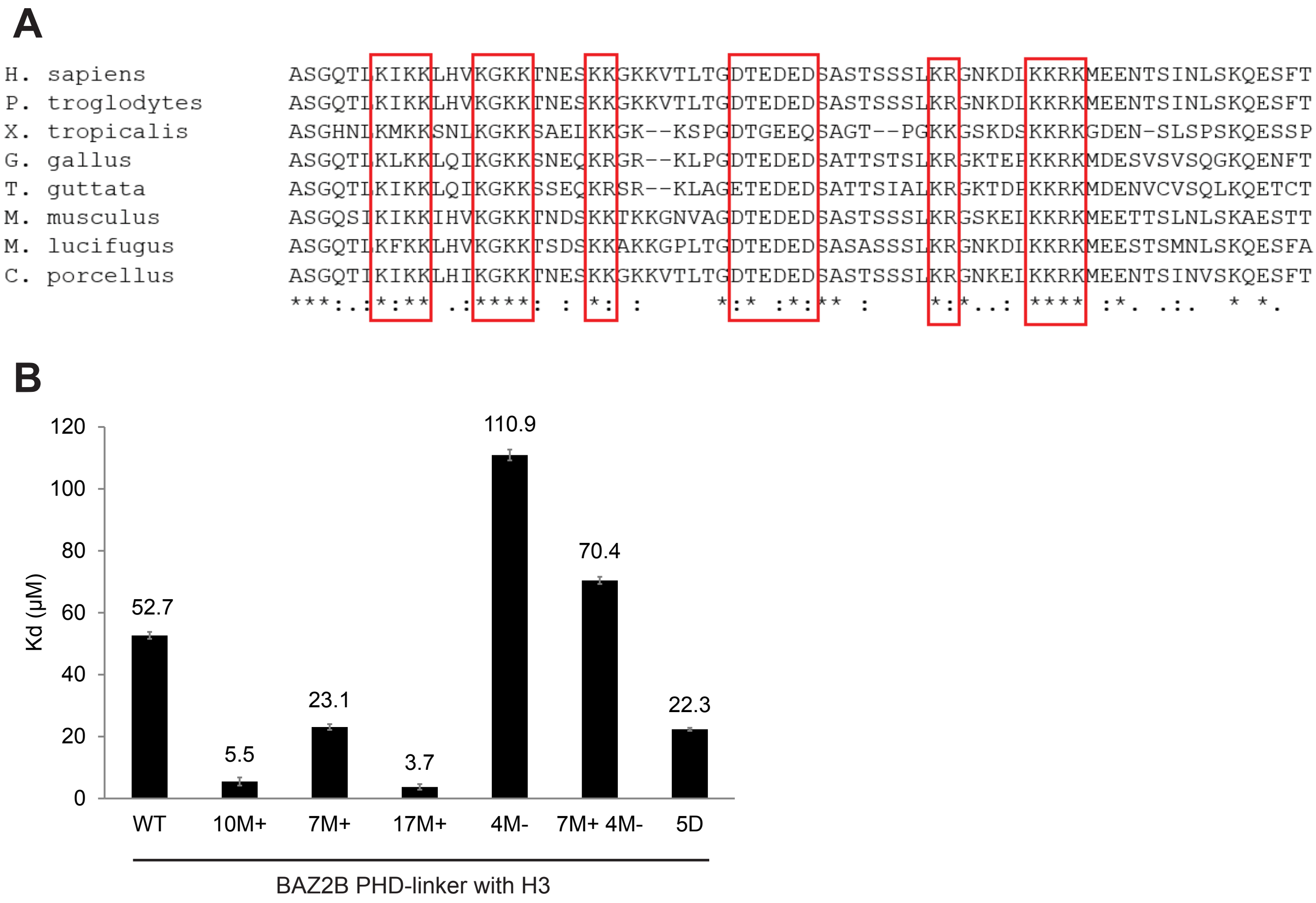

Supplemental Figure 7

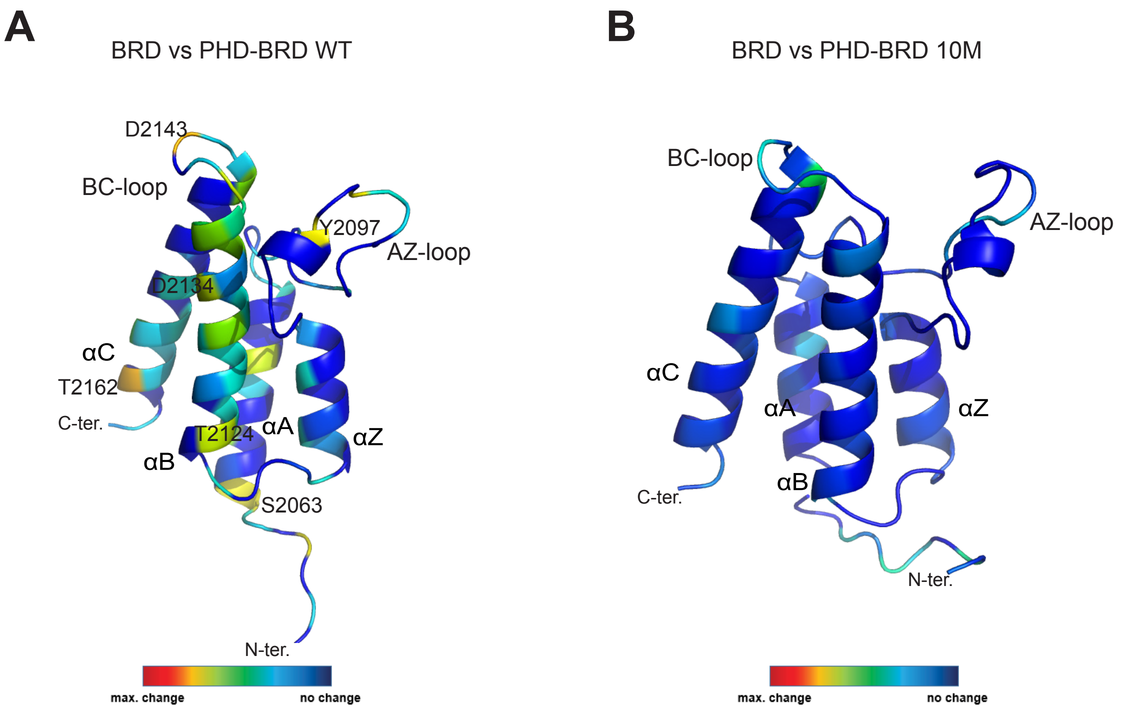

Supplemental Figure 8

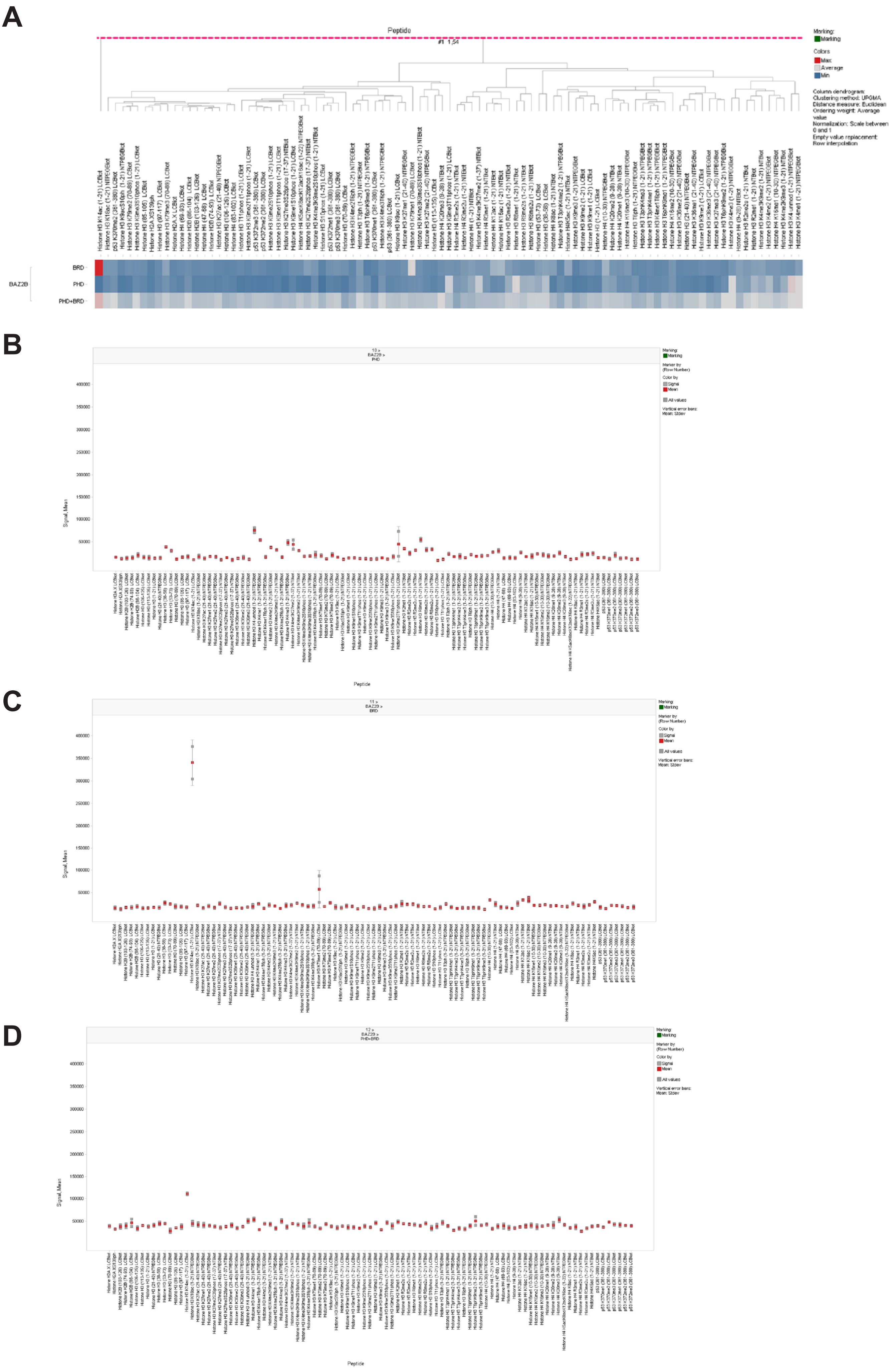

Supplement: Supplemental Data [file 10.1074_M117.801464_jbc.M117.801464-1.pdf]
